# GDNF-expressing macrophages mitigate loss of dopamine neurons and improve Parkinsonian symptoms in MitoPark mice

**DOI:** 10.1038/s41598-018-23795-4

**Published:** 2018-04-03

**Authors:** Cang Chen, Xiuhua Li, Guo Ge, Jingwei Liu, K. C. Biju, Suzette D. Laing, Yusheng Qian, Cori Ballard, Zhixu He, Eliezer Masliah, Robert A. Clark, Jason C. O’Connor, Senlin Li

**Affiliations:** 1 Department of Medicine, The University of Texas Health Science Center at San Antonio, 7703 Floyd Curl Drive, San Antonio, Texas 78229 USA; 2 Department of Pharmacology, The University of Texas Health Science Center at San Antonio, 7703 Floyd Curl Drive, San Antonio, Texas 78229 USA; 3 Stem Cells Research Center of Guizhou Medical University and Key Laboratory of Adult Stem cell Transformation Research, Chinese Academy of Medical Science, Guiyang, Guizhou 550025 China; 4 Division of Neurosciences, NIA-NIH, Bethesda, MD 20892, USA; 5 Audie L. Murphy VA Hospital, 7400 Merton Minter Boulevard, San Antonio, Texas 78229 USA

## Abstract

Glial cell line-derived neurotrophic factor (GDNF) is the most potent neuroprotective agent tested in cellular and animal models of Parkinson’s disease (PD). However, CNS delivery of GDNF is restricted by the blood-brain barrier (BBB). Using total body irradiation as transplant preconditioning, we previously reported that hematopoietic stem cell (HSC) transplantation (HSCT)-based macrophage-mediated gene therapy could deliver GDNF to the brain to prevent degeneration of nigrostriatal dopamine (DA) neurons in an acute murine neurotoxicity model. Here, we validate this therapeutic approach in a chronic progressive PD model – the MitoPark mouse, with head shielding to avoid inducing neuroinflammation and compromising BBB integrity. Bone marrow HSCs were transduced *ex vivo* with a lentiviral vector expressing macrophage promoter-driven GDNF and transplanted into MitoPark mice exhibiting well developed PD-like impairments. Transgene-expressing macrophages infiltrated the midbrains of MitoPark mice, but not normal littermates, and delivered GDNF locally. Macrophage GDNF delivery markedly improved both motor and non-motor symptoms, and dramatically mitigated the loss of both DA neurons in the substantia nigra and tyrosine hydroxylase-positive axonal terminals in the striatum. Our data support further development of this HSCT-based macrophage-mediated GDNF delivery approach in order to address the unmet need for a disease-modifying therapy for PD.

## Introduction

Parkinson’s disease (PD) is a prevalent chronic neurodegenerative disease characterized clinically by resting tremor, muscle rigidity, slowness of voluntary movement, and postural instability. It affects more than 1% of the global population aged 55 years and older^1,2^. PD is epitomized by a progressive loss of dopamine (DA) neurons in substantia nigra (SN) pars compacta (SNpc), leading to a DA deficit in the primary projection site, the striatum. The consequent dysregulation of basal ganglia circuits result in impairment of both motor and non-motor functions^3,4^. Currently, there is neither a cure for PD, nor any disease-modifying interventions^5^. With standard therapies, levodopa provides only symptomatic relief at early stages of PD, but fails to arrest the progressive loss of DA neurons. Further, this approach carries significant side effect liability, including dyskinesia and motor fluctuations, and eventually becomes ineffective^6^.

Glial cell line-derived neurotrophic factor (GDNF) is the most potent neuroprotective and neuroregenerative agent for the DA neurons affected in PD^7,8^. In neurotoxin-lesioned rodents and non-human primates, GDNF, delivered by direct brain injection, promotes dopaminergic neuronal survival and induces fiber outgrowth, while improving motor deficits^9–11^. However, GDNF does not cross the BBB, posing a substantial technical challenge for therapeutic application. To overcome BBB impermeability to GDNF, intermittent injections, continuous infusions, or genetically engineered cells released from capsules or injected focally have been employed, but these strategies have failed to achieve therapeutic efficacy^12–14^, largely due to either ineffective delivery of GDNF to the primary sites of neurodegeneration or the inability to cover large lesion areas in human brain. To overcome these limitations, we previously introduced a hematopoietic stem cell (HSC) transplantation-based macrophage-mediated GDNF delivery strategy^15^. This unique approach utilizes the macrophage property of homing to sites of neurodegeneration^16–18^. It also capitalizes on our highly active macrophage synthetic promoter (MSP)^19,20^, as well as efficient transduction of lentiviral vectors^21–23^. Using this model, either GDNF or neurturin (NTN) was effectively delivered to sites of neurodegeneration and dramatically ameliorated MPTP (1-methyl-4-phenyl-1,2,3,6-tetrahydropyridine)-induced loss of DA neurons in the SN and their terminals in the striatum^20,24^. However, MPTP-induced neurodegeneration features the acute loss of DA neurons and rapid onset of symptoms, thereby failing to model the characterstic chronic and progressive nature of PD. Moreover, the MPTP model is suitable only for testing preventive strategies, but not clinically relevant approaches to chronic progressive disease, such as cell-based gene delivery.

A genetically engineered murine model of PD – the MitoPark mouse – was reported in 2007^25^. In these animals, mitochondrial function is disrupted in DA neurons by selective deletion of the mitochondrial transcription factor Tfam^25^. Importantly, MitoPark mice exhibit the cardinal features of PD, including adult-onset neurodegeneration and progressive decline in motor and non-motor functions, as well as responsiveness to levodopa^25–27^. Therefore, the MitoPark mouse has emerged as an excellent model for studying PD etiology and testing therapeutic interventions^27–30^. In the present study, we utilized MitoPark PD mice to test the therapeutic efficacy of HSCT-based macrophage-mediated GDNF gene delivery. The results demonstrated that HSC-based macrophage delivery of GDNF effectively protected against dopaminergic neurodegeneration, resulting in significant reversal of both motor and non-motor dysfunction without adverse effects.

## Results

### MitoPark mice exhibited progressive loss of motor function

MitoPark mice or wild type normal control littermates were identified by genotyping (Suppl. 1a). Since progressive loss of motor function is a hallmark behavioral feature of MitoPark mice, spontaneous horizontal and vertical movements of MitoPark or normal control mice were recorded at different ages (Suppl. 1b,c). The significant decline in both horizontal and vertical activities of MitoPark mice became apparent by 12 weeks of age and progressed thereafter, for example decreasing to approximately 71% and 90%, respectively, compared with normal control mice.

### Lentiviral vector expressing GDNF protected the viability of MPP-treated SH-SY5Y cells

In order to validate *in vitro* the neuroprotective capacity of lentiviral vector-driven expression of GDNF, we measured the production of GDNF from vector-transfected murine macrophage cell line RAW 264.7 (Suppl. 2a) and used SH-SY5Y human neuroblastoma cells as a dopaminergic neuronal model to the assess GDNF-mediated cellular protection (Suppl. 2b). A very low concentration of GDNF (0.21 ± 0.06 ng/ml) was detectable in control GFP-transfected RAW 264.7 cell culture medium, whereas significantly higher secretion of GDNF protein was detected in RAW 264.7 cell cultures transfected with either hGDNF or rGDNF (8.36 ± 0.11 or 9.13 ± 0.29 ng/ml, respectively). MTT assay showed that conditioned media from GFP-transfected RAW 264.7 failed to preserve the viability of MPP^+^-treated SH-SY5Y cells, whereas conditioned media from either hGDNF or rGDNF transfected RAW 264.7 cell cultures increased MPP^+^-treated SH-SY5Y cell viability by 92.0% and 59.9%, respectively, compared with non-GDNF treated controls. Together, these data suggest that GDNF exerts a neuroprotective effect on SH-SY5Y cells.

### Macrophage-mediated GDNF therapy improved motor dysfunction in MitoPark mice

**Figure 1:**
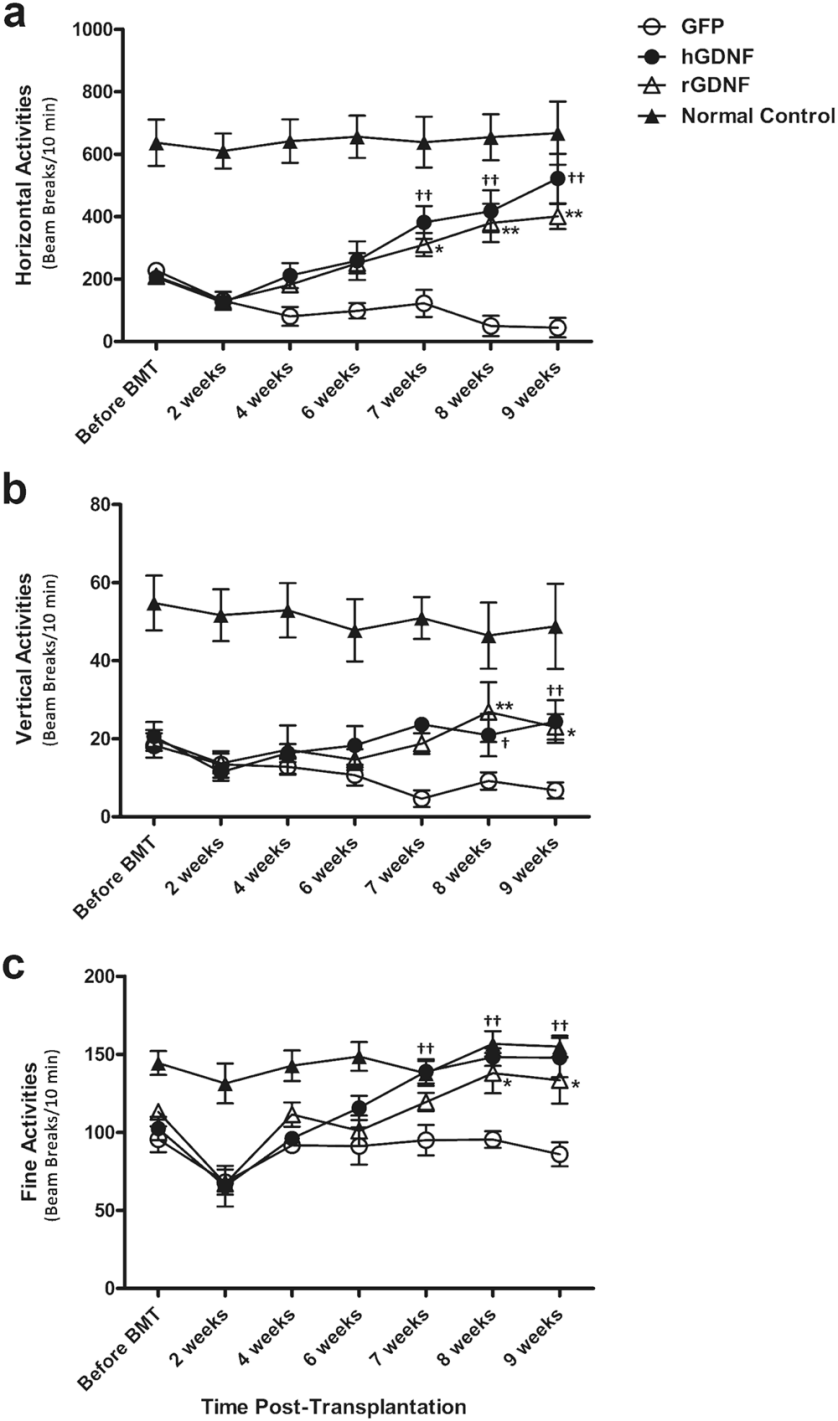
Effect of hematopoietic stem cell (HSC) transplantation-based macrophage-mediated GDNF delivery on locomotor activities in MitoPark mice. 18-week old MitoPark mice were transplanted with bone marrow cells which were transduced with lentiviral vectors expressing hGDNF, rGDNF or GFP cDNA, and a group of wild type normal control mice received GFP transduced bone marrow cell transplantation. The horizontal, vertical locomotor and fine activities including grooming, exploration on four limbs and sniffing were tested by the Photobeam Activity System (San Diego Instruments, San Diego, CA) before and after bone marrow transplantation (BMT) on each experimental group. The horizontal (**a**), vertical (**b**) locomotor activities and fine activities (**c**) were recorded for 60 minutes and assessed at 10-minutes time-bins. Each point represents mean of 6 × 10 minutes recorded from twelve animals per treatment group (n = 12). Error bars indicate ± SEM. Significant differences are indicated: *(for rGDNF), ^†^(for hGDNF) *P* < 0.05; **,^††^*P* < 0.01 versus GFP-transplanted MitoPark mice.

**Figure 2:**
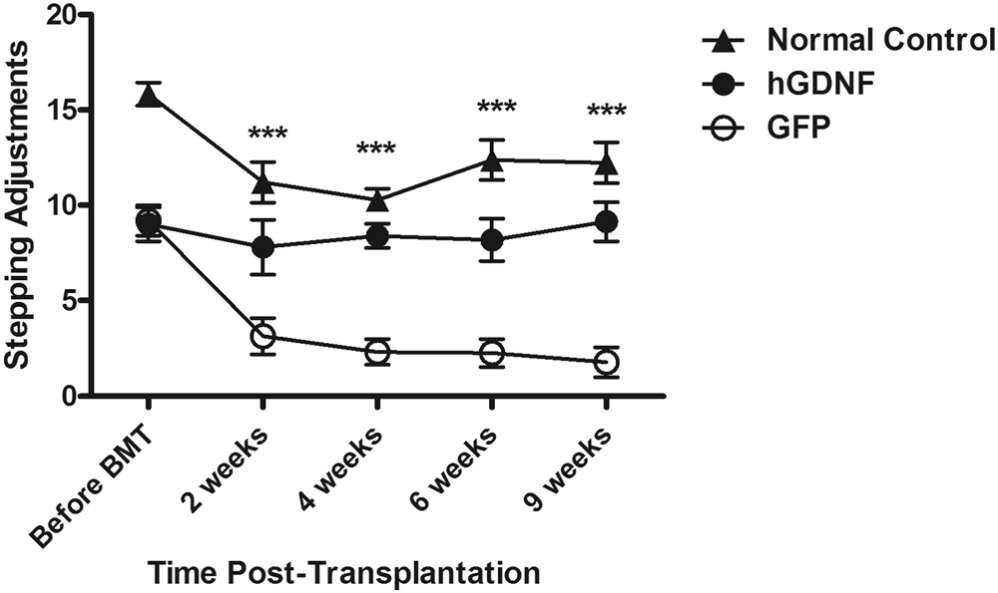
GDNF therapy improved the forelimb motor deficit in MitoPark mice. The forelimb motor performance was assessed by stepping test performed on MitoPark mice receiving hGDNF (n = 12) or GFP (n = 12) transduced bone marrow cell transplantation and wild type normal control mice receiving GFP transplantation (n = 12). Each point represents mean ± SEM from three trails on each animal per treatment group. Significant differences are indicated: ***P < 0.001, hGDNF-transplanted MitoPark mice versus GFP-transplanted MitoPark mice.

To assess the therapeutic effects of HSCT-based macrophage-mediated GDNF delivery on motor function in MitoPark mice, spontaneous horizontal (Fig. 1a) and vertical (Fig. 1b) activity, as well as fine movements (Fig. 1c), including grooming, exploration on four limbs and sniffing, were measured from 18 to 29 weeks of age before and after transplantation with gene-transduced cells. GFP-transplanted MitoPark mice exhibited a progressive decline in motor function. Their horizontal, vertical, and fine motor activities decreased by 75.6%, 63.1%, and 10.1% from baseline, respectively, at 9 weeks post-transplantation. In contrast, GDNF-transplanted MitoPark mice exhibited a steady improvement in their motor function with horizontal, vertical, and fine motor activities improved by 120.8%, 43.1%, and 53.5% from the baseline, respectively. Taken together, the horizontal, vertical and fine motor activities in the GDNF-transplanted groups reached 78%, 55%, and 94% of the levels in GFP-transplanted normal control mice at 9 weeks post-transplantation (Fig. 1a–c). In addition, forelimb motor function was decreased by 81% in GFP-transplanted MitoPark mice at 9 weeks post-transplantation, whereas the decline in forelimb motor function was fully mitigated in hGFNF-transplanted MitoPark mice (Fig. 2).

### Macrophage-mediated GDNF therapy improved nest building deficits in MitoPark mice

**Figure 3:**
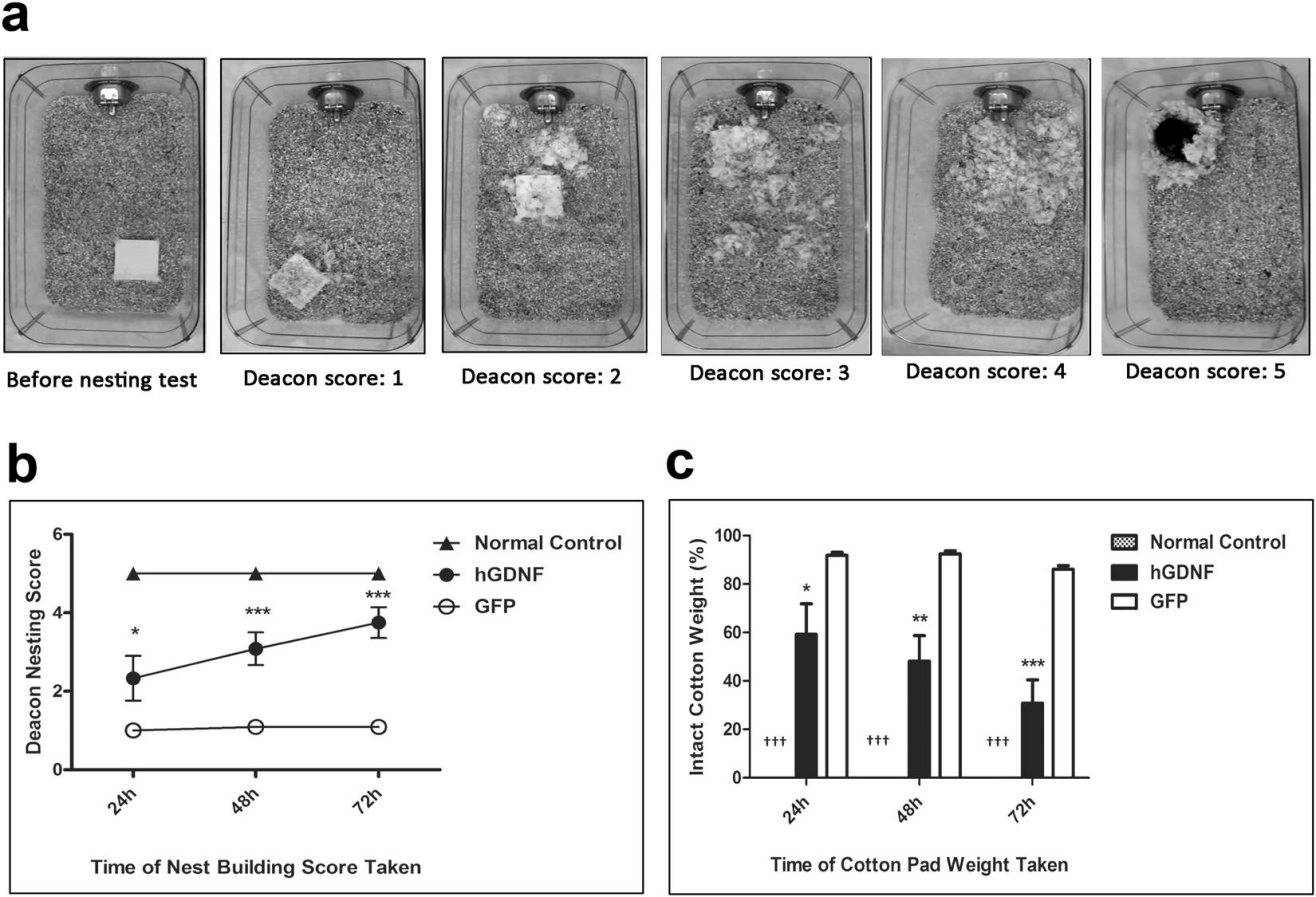
GDNF therapy improved the nest-building performance in MitoPark mice. (**a**) Assessment of the nest using Deacon’s scores of 1–5. Score 1: Nestlet not noticeably touched; 2: Nestlet partially torn up; 3. Nestlet mostly shredded but no identifiable nest site; 4. An identifiable, but flat nest; 5. A perfect nest with wall surrounded mouse body. (**b,c**) The nest building abilities of hGDNF-, GFP-transplanted MitoPark mice and GFP-transplanted wild type normal control mice were assessed by the nesting behavior test at 4 weeks post-transplantation. The nesting scores were taken at 24, 48 and 72 hours after a pre-weighted Nestlet™ cotton pad placed in each cage, and remaining unshredded piece were weighted at the same time. In (**b**) each point represents mean ± SEM from twelve animals per treatment group (n = 12). Significant differences are indicated: *P < 0.05, ***P < 0.001, hGDNF-transplanted MitoPark mice versus GFP-transplanted MitoPark mice. In (**c**) each bar represents mean ± SEM from twelve animals per treatment group (n = 12). *P < 0.05, **P < 0.01, ***P < 0.001 versus GFP- transplanted MitoPark mice. ^†††^*P* < 0.001 versus hGDNF- and GFP-transplanted MitoPark mice.

Deficits in nesting behavior, a motivated, goal-directed activity requiring orofacial and forelimb dexterity, correlate with striatal DA damage^31,32^. To quantify nesting behavior, we used Deacon’s five-point rating system (Fig. 3a,b) and measured the weight of unshredded nestlet material remaining at the indicated times (Fig. 3c) at 4 weeks post-transplantation. GFP-transplanted normal control mice built nests perfectly (score: 5) within 24 hours, leaving no unshredded pieces of nestlet. However, GFP-transplanted MitoPark mice failed to construct nests, even after 72 h. Their nesting score was low (1.09 ± 0.30) and 86.11 ± 1.52% of neslet remained unshredded, indicating severe impairment of nesting behavior. Notably, hGDNF-transplanted MitoPark mice exhibited a significant improvement in nesting behavior (Fig. 3b,c), achieving a nesting score of 3.75 ± 1.36 and leaving only 30.80 ± 9.65% of unshredded nestlet, notwithstanding their slower pace of nest building.

### Macrophage-mediated GDNF therapy improved anhedonia

**Figure 4:**
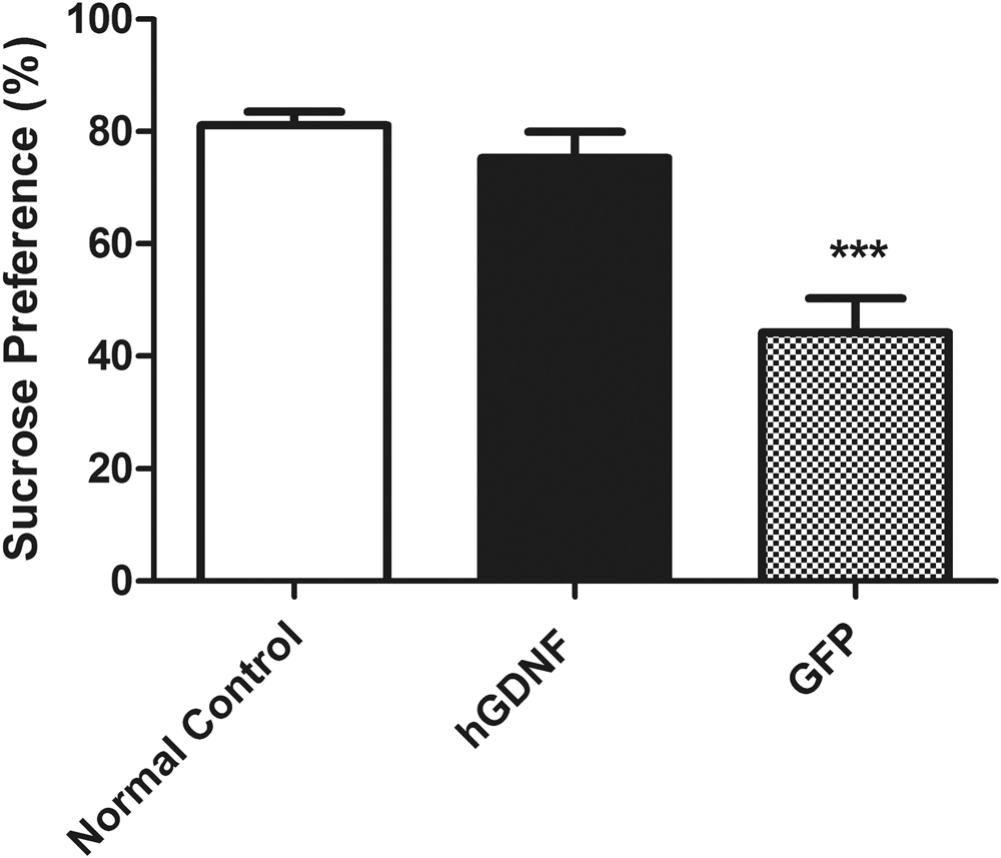
GDNF therapy improved the anhedoina associated with depression in MitoPark mice. Data were collected from the 4-day sucrose preference test. Each bar represents mean ± SEM from twelve animals per treatment group (n = 12). ***P < 0.001 versus hGDNF-transplanted MitoPark mice and GFP-transplanted wild type normal control mice.

To assess the therapeutic effects of GDNF delivery on depressive-like behavior in MitoPark mice, anhedonia-like behavior was measured using the sucrose preference test 6 weeks post-transplantation. Sucrose preference scores in GFP-transplanted control mice, hGDNF-, and GFP-transplanted MitoPark mice were 81.08 ± 2.45%, 75.27 ± 4.70%, and 44.20 ± 6.09%, respectively (Fig. 4). Thus, GFP-transplanted MitoPark mice exhibited an anhedonia-like phenotype, as indicated by significantly reduced sucrose preference compared with GFP-transplanted normal control mice. Notably, hGDNF treatment of MitoPark mice restored sucrose preference to 93% of normal control levels.

### Macrophage-mediated GDNF therapy protected against loss of DA neurons in SN and degeneration of dopaminergic terminals in striatum

**Figure 5:**
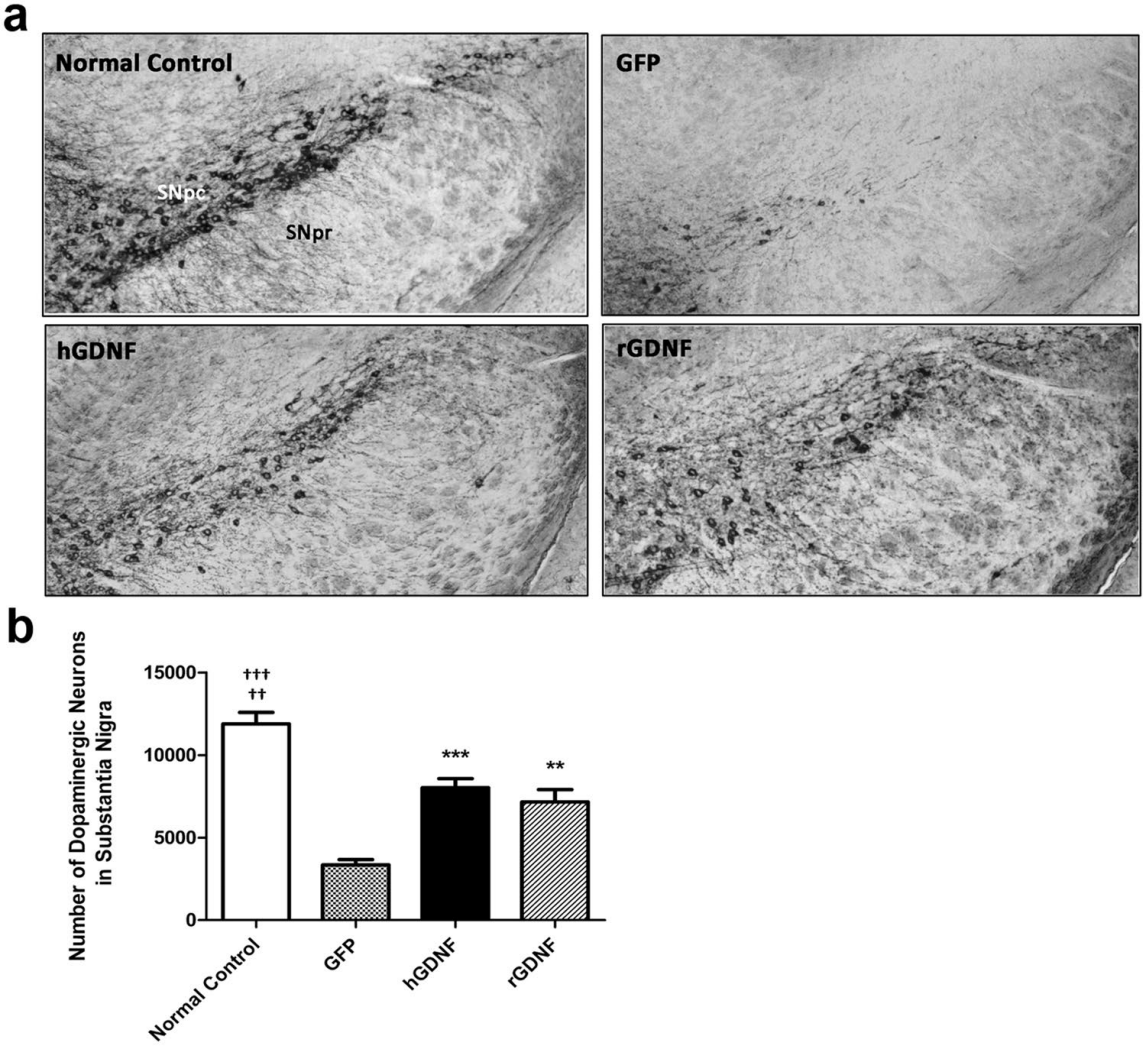
GDNF therapy protected loss of dopamine neurons in the substantia nigra of MitoPark mice. (**a**) 30 µm thick coronal midbrain sections were from randomly selected brain samples of hGDNF-, rGDNF-, GFP-transplanted MitoPark mice, and GFP-transplanted wild type normal control mice at 9 weeks post-transplantation. The sections were stained with tyrosine hydroxylase (TH) immunohistochemistry. Red stained TH-immunoreactive neurons are dopamine neurons distributed in substantia nigra at 100x magnification. SNpc, substantia nigra pars compacta; SNpr, substantia nigra pars reticulate. (**b**) The number of dopaminergic neurons in substantia nigra of hGDNF-, rGDNF-, GFP-transplanted MitoPark mice, and GFP-transplanted wild type normal control mice. The total number of Nissl^+^/TH-immunooreactive neurons were estimated by quantitative stereological analysis using the Zeiss AxioImager A1 microscope with Stereo Investigator software. Each bar represents mean ± SEM from six animals per treatment group (n = 6). **P < 0.01, ***P < 0.001, ^†††^*P* < 0.001 versus GFP transplanted-MitoPark mice. ^††^*P* < 0.01 versus hGDNF- and rGDNF-transplanted MitoPark mice.

**Figure 6:**
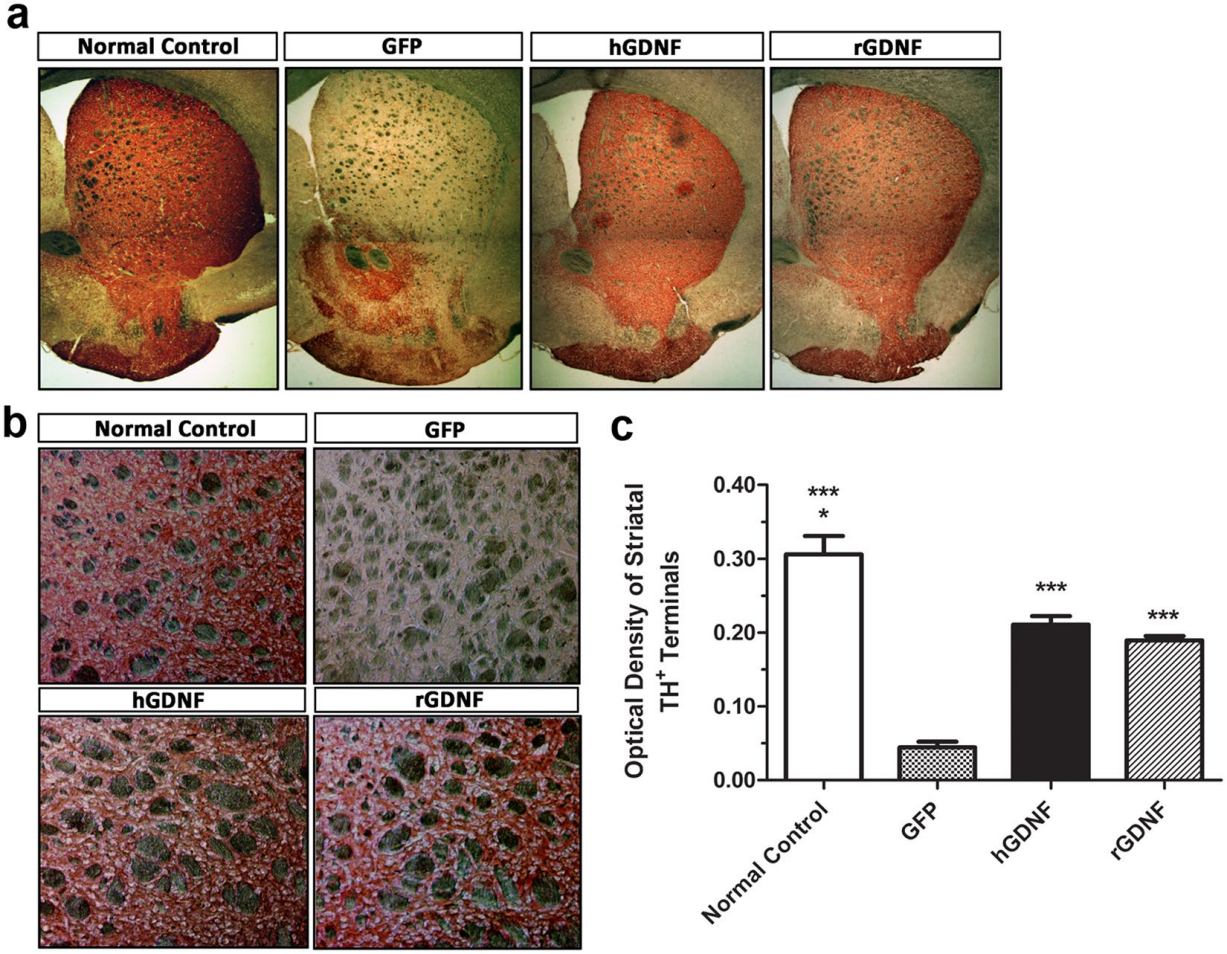
GDNF therapy protected degeneration of dopaminergic terminals in the striatum of MitoPark mice. (**a**) 30 µm thick coronal forebrain sections were from randomly selected brain samples of hGDNF-, rGDNF-, GFP-transplanted MitoPark mice, and GFP-transplanted wild type normal control mice at 9 weeks post-transplantation. The sections were stained with tyrosine hydroxylase (TH) immunohistochemistry, and TH-immunoreactive dopaminergic terminals in the striatum were stained with red. At 20x magnification. (**b**) High power photomicrograph of TH staining in the dorsolateral striatum. At 100x magnification (**c**) Optical densities of striatal TH^+^ terminals were measured from digitized images of the striatum using NIH ImageJ software. Each bar represents mean ± SEM from six animals per treatment group (n = 6). *P < 0.05 versus hGDNF- and rGDNF-transplanted MitoPark mice, ***P < 0.001 versus GFP-transplanted MitoPark mice.

At 9 weeks after transplantation, the neuroprotective effects of macrophage-mediated GDNF delivery on the nigrostriatal dopaminergic system were assessed by quantitative stereological analysis of tyrosine hydroxylase-positive (TH^+^) neurons in the SN pars compacta (SNpc), as well as by assessment of the density of TH^+^ terminals in the striatum. We detected 11,883 ± 1,707 TH^+^ neurons in SNpc from GFP-transplanted normal control mice (Fig. 5). Relative to this benchmark, GFP-transplanted MitoPark mice exhibited up to a 72% loss of TH^+^ neurons in SNpc, whereas hGDNF- or rGDNF-treated MitoPark mice exhibited only a 33% or 39% loss, respectively. In addition, the TH^+^ dendritic fiber networks in the SNpr were also reduced in the GFP-transplanted MitoPark mice, but preserved in hGDNF- and rGDNF-treated MitoPark mice (Fig. 5). Similarly, the neuroprotective effects of GDNF delivery were apparent in the striatum of hGDNF- or rGDNF-treated MitoPark mice. A higher intensity of TH-immunostained dopaminergic terminal fibers in the striatum was observed in GFP-transplanted normal control mice, whereas a lower intensity was found in the GFP-transplanted MitoPark mice. The hGDNF- or rGDNF-transplanted MitoPark mice displayed significantly preserved intensity of TH^+^ stained terminals in the striatum (Fig. 6a). Under high magnification, we observed many thick TH^+^ fibers with branches in the striatum of hGDNF- and rGDNF-transplanted MitoPark mice, but not GFP-transplanted MitoPark mice, suggesting a regenerative response of the dopaminergic terminals to the GDNF therapy (Fig. 6b). To quantify the intensity of TH-stained terminals, optical density was measured at the dorsolateral aspects of the striatum, the region receiving the largest share of innervation from DA neurons in SNpc (Fig. 6c). Relative to the normal control mice, a significant reduction (~85%) in TH^+^ terminals was observed in the striatum of GFP-transplanted MitoPark mice, whereas the optical density of striatal TH^+^ terminals in hGDNF- or rGDNF-transplanted MitoPark mice was only 31% or 37% lower, respectively, than in GFP-transplanted normal control mice.

### Transgene expression was observed in macrophages after HSC transplantation

To assess the efficacy of bone marrow HSC transplantation, GFP expression in peripheral blood leukocytes of the GFP-transplanted MitoPark mice was analyzed by fluorescence-activated cell sorting (FACS) at 8 weeks post-transplantation. There were 90.03 ± 0.02% GFP-positive (GFP^+^) blood leukocytes detected in positive control GFP-transgenic mice and 64.82 ± 1.40% GFP-expressing cells in Lenti-MSP-GFP-transplanted MitoPark mice (Suppl. 3a). In the Lenti-MSP-GFP-transplanted MitoPark mice, 71.64 ± 6.55% of CD11b- (monocyte/macrophage marker) positive (CD11b^+^) leukocytes expressed GFP with high fluorescence intensity, whereas only 8.44 ± 1.34% of CD11b-negative (CD11b^−^) leukocytes expressed GFP (Suppl. 3b), and GFP intensity was significantly lower in CD11b^−^ cells (Suppl. 3c). These data suggest that MSP was driving the expression of the transgene selectively in cells of the monocyte/macrophage lineage.

### Gene-modified macrophages/microglia were selectively recruited to the areas of neurodegeneration in SN

**Figure 7:**
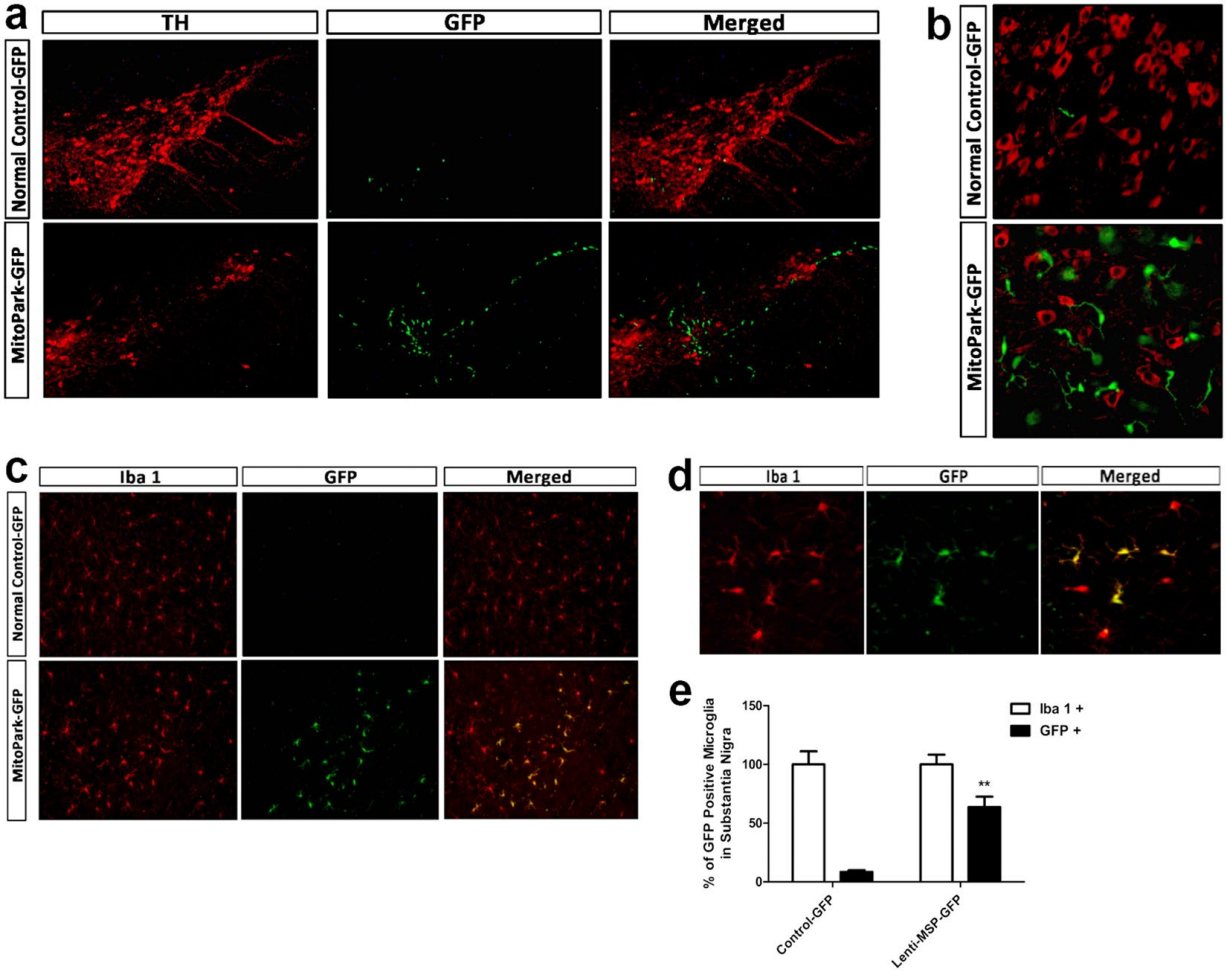
Gene-modified macrophages/microglia are recruited to the dopamine neuron loss area in substantia nigra following bone marrow transplantation. (**a**) Midbrain sections from GFP-transplanted wild type normal control mice and MitoPark mice were stained with tyrosine hydroxylase (TH) fluorescent immunohistochemistry at 9 weeks post-transplantation. At 100x magnification (**b**) Representative high power photomicrograph of TH staining in the nigra. At 200x magnification (**c**) Iba 1-positive (red) microglia expressing GFP (green) in the substantia nigra of GFP-transplanted MitoPark mice. At 100x magnification (**d**) High power photomicrograph of Iba 1-stained microglia including infiltrated GFP-expressing microglia at 200x magnification (**e**) Proportion of bone marrow-derived GFP positive (green) microglia in the nigra. Each bar represents mean ± SEM from five animals per treatment group (n = 5). **P < 0.01 versus GFP-transplanted wild type normal control mice.

To verify that macrophages derived from gene-modified bone marrow HSCs infiltrated into the sites of DA neuron degeneration in the recipient mice after transplantation, we examined midbrain sections from GFP-transplanted MitoPark or normal control mice stained with TH or Iba-1 (microglia/macrophage marker) by fluorescent immunohistochemistry. As shown in Fig. 7a, GFP-transplanted normal control mice had abundant TH^+^ DA neurons (red) in the SN, with very few GFP-expressing cells (green) observed. In contrast, GFP-transplanted MitoPark mice manifested a clear loss of DA neurons at SN and a robust infiltration of GFP-expressing macrophages into the area of DA neuron loss. Of particular note, high-power photomicrography of TH-fluorescent staining in the SN of GFP-transplanted MitoPark mice demonstrated that the genetically modified bone morrow-derived microglia were in close proximity to degenerating DA neurons (Fig. 7b). In Fig. 7c, the midbrain sections from GFP-transplanted control mice showed that Iba 1-positive (Iba 1^+^) cells in the SN were abundant (red), whereas GFP-expressing transplanted cells were rare. In contrast, most of the Iba 1^+^ cells in the SN of GFP-transplanted MitoPark mice were also positive for GFP, and these cells were distributed throughout the area of DA neuron loss (Fig. 7c,d). Quantification demonstrated that 63.5 ± 6.26% of Iba 1^+^ cells expressed GFP in GFP-transplanted MitoPark mice, whereas only 8.1 ± 1.58% were GFP^+^ in GFP-transplanted normal control mice (Fig. 7e). GFP^+^ cells were not found in other brain regions such as thalamus or cortex (data not shown), suggesting specific homing of transplanted cells to sites of neurodegeneration in the brain of the MitoPark mice.

### GDNF was detected in plasma, SN, and striatum after HSC transplantation

**Figure 8:**
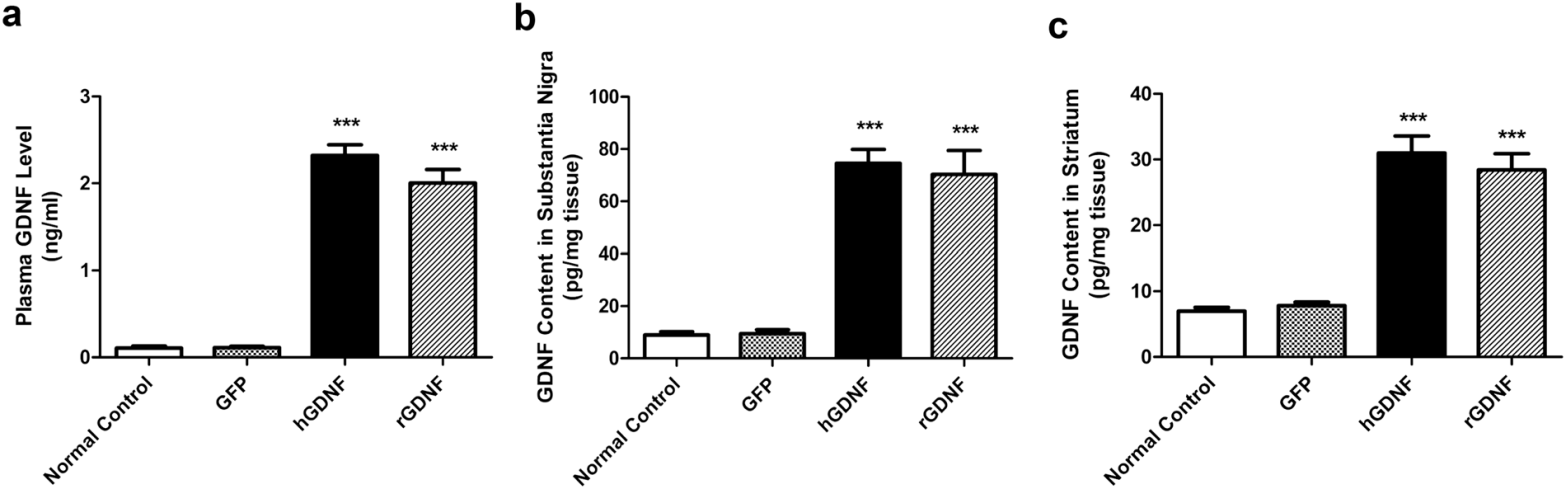
GDNF levels in plasma, substantia nigra and striatum following bone marrow transplantation. The plasma samples, forebrain and midbrain tissues were collected from nine weeks post-hGDNF-, rGDNF- or GFP-transplanted MitoPark mice and wild type normal control mice. (**a**) The plasma GDNF levels, (**b**) substantia nigra GDNF content and (**c**) striatum GDNF content were determined by enzyme-linked immunosorbent assay. Each bar represents mean ± SEM from eight animals per treatment group for plasma (n = 8) and from five animals per treatment group for substantia nigra and striatum tissues (n = 5). ***P < 0.001 versus GFP-transplanted MitoPark mice and wild type normal control mice.

Plasma samples, as well as forebrain and midbrain tissues, were collected at the termination of the experiment, 9 weeks after hGDNF-, rGDNF- or GFP-transplantation, and GDNF levels were determined by enzyme-linked immunosorbent assay. As shown in Fig. 8a, plasma GDNF levels in hGDNF- and rGDNF-transplanted MitoPark mice were 2.321 ± 0.122 and 2.004 ± 0.155 ng/ml, significantly higher than those detected in GFP-transplanted MitoPark and normal control mice. Similarly, the GDNF levels in SN from hGDNF- and rGDNF-transplanted MitoPark mice was 74.564 ± 5.336 and 70.314 ± 9.155 pg/mg tissue, respectively, reflecting 7.89-fold and 7.44-fold enrichment relative to GFP-transplanted MitoPark mice (Fig. 8b). The GDNF content detected in the striatum of hGDNF- and rGDNF-transplanted MitoPark mice was 30.979 ± 2.605 and 28.418 ± 2.465 pg/mg tissue, respectively, reflecting 3.97-fold and 3.64-fold enrichment compared to GFP-transplanted MitoPark mice (Fig. 8c).

### No evidence of side effects of macrophage-mediated GDNF therapy was detected

Previous reports indicated several adverse effects of GDNF infusion approaches, such as allodynia and weight loss, pancreatic and myocardiocyte lesions^33^. GDNF also acted on non-neural organ systems, such as the kidneys and testes^34^. Therefore, we monitored mouse body weight, measured cold allodynia, and examined H&E-stained tissue sections from the kidneys, testes, pancreas, and heart for pathological assessment after the cell-based GDNF treatment. As shown in Suppl. 4a and b, no increase in topical acetone-induced response frequency or time extension of paw lift in the GDNF-transplanted MitoPark or normal control mice compared with GFP-transplanted mice. Body weight was recorded weekly before and after transplantation (Suppl. 4c). GFP-transplanted MitoPark mice exhibited a progressive decrease in body weight with age. However, body weight was maintained in GDNF-transplanted MitoPark mice and GFP-transplanted normal control mice. Histological examination of the kidneys and testes from GDNF-treated mice revealed no morphologic abnormalities in comparison with normal control mice (Suppl. 5a and b). Moreover, no tissue pathology was observed in the pancreas (e.g., acinar to ductular metaplasia) or in the heart (e.g., multifocal degeneration with interstitial fibrosis) due to GDNF therapy (Suppl. 5c and d).

## Discussion

Our study demonstrates significant monocyte/macrophage homing to areas of neurodegeneration in the MitoPark PD model, with the infiltrating macrophages settling in close proximity to degenerating neurons. The trafficking/homing of these cells occurs without compromising the BBB, as head shielding was used during the pre-treatment irradiation. The macrophages were pre-programmed to express and secrete GDNF via lentiviral HSC gene therapy, thus comprising an effective cellular vehicle for overcoming BBB limitations for delivery to degenerating neurons. Macrophage-mediated GDNF delivery dramatically ameliorates loss of TH^+^ neurons of the SN and TH^+^ terminals in the striatum and thereby results in profound mitigation of motor as well as non-motor parkinsonian symptoms.

### CNS delivery by macrophages – hematopoietic stem cell transplantation–based versus intravenous infusion of macrophages

These data are consistent with our previous studies and a recent report showing in a 6-OHDA-intoxicated mouse model that systemically infused GDNF-transfected M2 macrophages home to the inflamed brain and produce potent neuroprotective effects as assessed by brain histochemical and behavioral tests^35^. Although we did not classify macrophage subtypes in the present study, it is unlikely that M2 macrophages possess an overall advantage over M1 macrophages in inflammatory trafficking/homing. Due to the acute nature of the 6-OHDA-intoxication model, short-term (21 days) neuroprotection was observed^35^. We speculate that periodic administration of the therapeutic cells might be required in this infusion (vs. transplantation), given the chronic nature of human PD, the relatively short lifespan of macrophages, and the dynamic properties of macrophage homing to the CNS. Although monocytes isolated by MACS and/or FACS can be transduced and transplanted, the feasibility and availability of large amounts of genetically engineered autologous monocytes for long-term sustained delivery of GDNF remains uncertain. Although the utility of monocytes as a CNS gene delivery vehicle has been shown in an Alzheimer’s disease model, the authors noted that bone marrow stem cells would be a better option^18^. HSC gene therapy for X-SCID patients exhibits sustained immune reconstitution over at least 12 years. Once HSCT/BMT is done successfully, engrafted donor HSCs continuously repopulate all blood cell lineages including monocytes/macrophages. The newly generated macrophages would carry genetic instruction for expression and secretion of GDNF accessible to neighbor neurons when they home to the CNS. We speculate that this process would be going on as long as the neurodegeneration is not resolved. However, this has not yet been fully investigated. A combination of multiple time point survey after HSCT and/or single macrophage infusion would be warranted to provide evidence.

However, some previous studies demonstrated that long-term GDNF over-expression can lead ultimately to decreases in dopamine levels via down-regulation of TH expression^36,37^, although in these studies the levels of GDNF in SN (2.5–4.5 ng/mm)^36,37^ were much higher (~50-fold) than in our approach (0.07 ng/mg). The lowest GDNF levels leading to suppressed dopamine were about 0.4 ng/mg^37^, 5-fold higher than the concentration we had, suggesting that our approach would provide a safe GDNF level.

### Microglia arise from monocytes/macrophages with or without brain conditioning

Our data may provide insight into two extant questions: 1) whether monocytes/macrophages can selectively home to a pathological brain region without disruption of the BBB; and 2) whether this alternative approach offers a viable therapeutic strategy in light of the reported failures of focal GDNF delivery for Parkinson’s disease. Genetic tracing and fate mapping in the mouse clearly demonstrate that microglia colonize developmentally during embryogenesis and are not replaced from bone marrow cells during healthy adult life^38–41^. In some mouse models that lack disruption of BBB, adult brain derivation of microglia from monocytes occurred only under certain conditions (e.g., tissue irradiation), highlighting the caveat regarding early irradiation-based bone marrow chimera study results^41^. However, not all situations fit this mold, as carefully designed studies demonstrated that conditioning is not always essential. For example, both *i. v*. injected and endogenous monocytes/macrophages infiltrated the CNS without conditioning in models of certain neurodegenerative diseases^18,42–44^ and psychological stress^45,46^. We and others^47^ have shown that monocyte-derived macrophages infiltrated the brain parenchyma of the SN of MPTP-treated mice conditioned for transplantation by head-protected irradiation, as is the case in the present study using head–shielded MitoPark mice. Infiltrating monocytes/macrophages accounted for up to 70% of Iba1^+^ macrophage/microglia in the SN. Therefore, we argue that monocyte/macrophage infiltration is likely the predominant mechanism for maintenance of nigral microglia or microglia-like cells in PD mouse models and this mechanism can be utilized to deliver GDNF or other neurotrophic factors to the diseased region to protect neurons from further degeneration. Whether this result will apply to PD patients remains to be determined. However, the success of clinical trials of lentiviral HSC gene therapy for X-Linked adrenoleukodystrophy (ALD)^48^ and metachromatic leukodystrophy (MLD)^23^, in which macrophages/microglia are believed to be the effector cells providing the missing proteins in the patients’ CNS, raise expectations of positive answers. Nonetheless, the issue was again complicated by transplant pre-conditioning that might compromise the BBB. A carefully designed study without non PD-related BBB damage may be warranted in non-human primates. Treosulfan, a BBB-impenetrable chemotherapeutic agent used in pre-conditioning, may be helpful in this effort.

An alternative mechanism has been proposed for donor cell infiltration into the brain, namely a short-term (within a few days of transplantation) wave of brain infiltration by the infused/transplanted HSCs occurs, independently of both the preparative regimen and the presence of a disease state in the brain^49^. In this scenario, it would be the early cellular immigrants that contribute to the turnover of microglia, rather than translocation of mature cells from the circulation. The turnover of microglia in this model took place only upon the use of a conditioning regimen capable of ablating functionally defined brain-resident myeloid precursors^49^. CNS infiltration of i. v. injected macrophages in a mouse model of Alzheimer’s disease did not support this theory^18^. Although our experimental setting did not allow us to compare these two mechanisms, the brain infiltration of monocytes/macrophages did not depend on any conditioning regimen that ablated brain-resident myeloid precursors and/or microglia because the heads of the recipient mice were protected from irradiation by entire head coverage with lead shields. Nonetheless, the HSCT-based therapy does provide the basic component (mega doses of HSC infusion) for a short-term wave of HSC brain infiltration to happen.

For lentiviral HSC gene therapy to be effective in neurodegenerative disorders, whatever the underlying mechanism may be, primary therapeutic cells must reach the affected SN within close proximity to degenerating neurons. Promising clinical benefits after lentiviral HSC gene therapy were first reported in patients with ALD^48^ and later in those with MLD^23^. The major contrast is that in the leukodystrophies, the therapeutic gene product for macrophage delivery is a peroxisomal or lysosomal protein missing in the patients, whereas in Parkinson’s disease, the therapeutic gene product for macrophage delivery is an over-expressed neurotrophic factor. The GDNF secreted by the macrophages/microglial cells functions as ligand for its receptor on the neuronal cell membrane, which is expected to be at least as efficient as cross-correction – a mechanism relevant to atypical secretory proteins in ALD and MLD. Besides being a typical secretory protein, GDNF is released in exosomes by macrophages^35^, a mechanism that could be involved in our intervention.

### Effective GDNF delivery to CNS – brain injection/infusion versus macrophage-mediated

Failures of the latest clinical trials using focal delivery of either recombinant protein or viral vectors of GDNF (or neurturin) dampen the enthusiasm of many investigators for neurotrophic factors as PD therapeutics, as recent failed trials applied improved delivery methods and selected early to middle stage PD patients (GDNF, Parkinsons UK)^13^. However, an in-depth analysis of the problem indicated that the failure of focal delivery to achieve the primary goal was not surprising, considering that convection-enhanced delivery (CED), a key component of these clinical trials, has been generally unsuccessful for a number of CNS diseases, including malignant gliomas^50^. The human putamen is simply too large for coverage by one or two delivery catheters, even though a similar approach may be effective in monkeys having brain size about one 15^th^ that of humans. The CED technology is limited in delivery efficiency/power^51,52^. In contrast, infiltrating macrophage-based GDNF brain delivery can be extremely effective regardless of the target size. This is because monocytes/macrophages may infiltrate throughout specific areas of neurodegeneration. In the SNpc of chronic Parkinsonian macaques, microglial cell processes and cell bodies establish contacts with dopaminergic neurites and cell bodies^53^. A recent special issue of *Science* vividly illustrates the intimate contacts of microglial cells with surrounding neurons – one microglial cell can touch several neurons, and a neuron can be reached by several microglia cells^54^. When these macrophages/microglial cells are genetically engineered to secrete GDNF protein, as they do in our study, one can envision them as an ideal cellular vehicle for selective delivery of GDNF to the cells that require trophic support the most. We consistently observed 5- to 10-fold increases in total nigral GDNF levels using this approach, even though BBB permeability was unchanged across groups.

### HSCT-based macrophage-mediated GDNF gene therapy for PD is both disease-modifying and symptom-improving

The present study extends our unique HSCT-based macrophage-mediated GDNF gene therapy strategy to a more clinically relevant animal model of PD, the MitoPark mouse, with adult onset and progressive disease manifestations. MitoPark mice exhibit the onset of significant reductions in locomotor activities at ~12 weeks of age with continuing decline over the next 4 to 5 months, thus providing a window of ~30 weeks to monitor the progression of PD-like symptoms and evaluate the efficacy of therapeutic interventions^28^. We chose to start the intervention at 18 weeks of age, as the animals have established motor as well as non-motor dysfunction at this time, mimicking the mid-stage of human PD.

We demonstrate here that HSCT-based GDNF gene therapy significantly improved motor deficits in the MitoPark mouse model. The therapeutic effects of GDNF on horizontal, vertical, and fine motor activities became significant by 7 to 8 weeks after HSCT. Our results are in agreement with a recent report that surgical intrastriatal delivery of adeno-associated virus (AAV) vector expressing rat GDNF can increase locomoter performance and alleviate the progressive motor dysfunction in MitoPark mice^55^. Forelimb akinesia in animal models, as assessed by the stepping test, is an important parameter thought to resemble limb akinesia and gait problems often seen in patients with PD^56^. In rats, the degree of impairment in stepping performance is correlated with the nigrostriatal DA lesion^57^. L-DOPA administration significantly improved stepping performance in MPTP-treated mice, but the reversal effect was no longer apparent 24 hours post-injection^56^. In our experiment, GDNF gene therapy significantly increased the number of adjusting steps and maintained therapeutic benefit on forelimb motor deficits in the MitoPark mice throughout the 9 week time period after HSCT.

PD is a neurodegenerative disorder characterized not only by motor dysfunction, but also by various non-motor symptoms, including impaired executive function and depression, which contribute significantly to disability in PD patients^3,58^. Therefore, we assessed nest-building ability and anhedonia-like behavior. MitoPark mice were severely compromised in the nest-building task at 22 weeks of age, whereas macrophage-mediated GDNF therapy significantly improved nesting behavior in these animals at 4 weeks after HSCT. Nesting behavior is considered to be a goal-directed activity involving stereotyped sensorimotor actions^59^ and requiring orofacial and forepaw performance, which is very sensitive to striatal DA damage^60^. However, the nest-building deficits could not be reversed by L-DOPA in MPTP-treated mice^61^. Taken together, the favorable effects of GDNF on nest-building deficits that we observed may involve other neurotransmitters in addition to the dopaminergic pathway and reflect a combination of motor and motivational deficits.

To assess the affective state, the sucrose preference test was performed at 6 weeks post-HSCT. GDNF significantly increased sucrose preference in MitoPark mice, suggesting a reversal of anhedonia-like behavior. The etiology of depression in PD is complex, and the exact mechanism underlying the effect of GDNF on depression is elusive. The dopaminergic and serotoninergic systems may play central roles, although the ventral tegmental area-nucleus accumbens pathway may be also involved^62,63^.

Our previous preventive studies demonstrated that macrophage-mediated GDNF or NTN treatment dramatically ameliorated MPTP-induced degeneration of TH^+^ neurons of the SN and TH^+^ terminals in the striatum, and stimulated axon regeneration^20,24^. In the present study, GDNF-treated mice revealed a 218–239% or 420–460% increase in survival of nigral DA neurons or striatal DA fibers compared with GFP-treated mice. These results are not only in agreement with previous reports demonstrating the neuroprotective effects of focal delivery of GDNF in rats and primate models of PD^9,11,64^, but also support the concept that transgene-expressing macrophages/microglia can serve as cellular vehicles^65^ for CNS delivery of therapeutic factors, including GDNF. Out of all TH-stained midbrain sections from normal control-GFP mice, we occasionally observed a few GFP-expressing macrophages in SN. This might be attributable to brain microvessel defects or local inflammation due to unknown reason. Intraneuronal inclusions in SN of MitoPark mice were observed by electron microscopy in our preliminary study. It will be interesting to determine in future studies whether our therapeutic intervention reduces the number and size of the intracellular inclusions.

### Safety considerations

Infection and catheter misplacement were reported as postoperative complications in the double-blind placebo-controlled study of stereotactic injection of GDNF. Allodynia and weight loss were also reported to be side effects of brain infusion of GDNF^33^. Moreover, GDNF exerts biological effects outside of the CNS, acting as a kidney morphogen during embryonic development and regulating the differentiation of spermatogonia in the testis^34^. Relative adverse effects might be induced, particularly by sustained, high-level expression of this biologically active molecule in the human brain^66^. A non-human primate study showed that three of five animals that received high doses of GDNF developed an unusual pattern of cerebellar toxicity^13^ characterized by focal lesions with a limited loss of Purkinje cells and near-complete loss of granule and molecular cell layers. However, no such adverse effects were observed in our experimental animals. Nevertheless, the lentiviral-transduced HSC-based macrophage-mediated GDNF delivery may have unique safety concerns. The integration of lentiviral vectors and gene expression cassettes into the genome of HSCs would be random and possibly close to oncogenes, thus leading to a risk for tumorigenesis. This was demonstrated in early retroviral HSC IL2RG gene therapy for X-SCID patients, in which 4 of the 10 treated patients eventually developed leukemia, likely due to LMO2 activation induced by integration of the retrovirus and cooperation between the functions of LMO2 and IL2RG^67^. Cell clone over-expansion also occurred in CGD patients treated with retroviral HSC gene therapy^68^. However, similar therapy did not cause cancerous clone expansion in ADA-SCID patients^69^. Collectively, retroviral integration-induced tumorigenesis may be gene specific. GDNF is not known to stimulate blood cell growth or development, and we did not observe any signs of blood malignancy in our study. Whether GDNF can lead to tumorigenesis in such a setting certainly warrants systematic further investigation. Unlike retroviruses, lentiviral vectors do not have a preference for inserting into promoter regions^70^. So far, lentiviral vector-based HSC gene therapy has enjoyed a rather safe profile regarding integration-induced tumorigenesis. Nevertheless, the latest genome-editing technologies, CRISPR/Cas9 in particular, provide great tools for avoiding random integration-related tumorigenesis^71–73^. Therapeutic GDNF cassettes can be inserted into a safe harbor (such as AAVS1) of the genome using these technologies.

Another safety issue arose from HSCT. For HSCT to work, recipients have to be preconditioned to empty the bone marrow stem cell niches, classically achieved by chemotherapy and/or whole body irradiation. Unfortunately, this conventional pre-transplant conditioning is quite toxic, leading to severe short-term and long-term adverse effects including death, and thus is reserved only for life-threatening conditions. Scientists and physicians have been searching for gentle pre-conditioning regimens, but without breakthrough success until very recently^74,75^. The two reported pre-conditioning regimens result in high and persistent transplantation efficiency, independent of chemotherapy and irradiation. However, they were still somewhat cytotoxic/cytocidal. Our group has also recently conceptualized and established completely noncytotoxic pre-conditioning-based HSCT in mice (unpublished). Although these new technologies require further investigation, they may one day lead to next generation of HSCT and well justify their applications in treatment of a chronic disease that is not immediately life threatening such as PD.

In conclusion, our data demonstrate that combined HSCT-based macrophage-mediated GDNF gene therapy effectively delivers the therapeutic factor to sites of neurodegeneration and protects against loss of dopaminergic neurons and projection terminals in the MitoPark mouse model of PD, even when the intervention was initiated after the animals had manifested fully developed PD-like symptoms. GDNF therapy not only reduced the progressive motor deficits, but also ameliorated non-motor dysfunction. Our novel approach may help to realize the neuroprotective potential of GDNF and lead to a unique disease-modifying treatment for PD patients.

## Methods

### Animals

MitoPark mouse breeding pairs were obtained from Dr. Nils-Göran Larsson (Karolinska Institutet, Stockholm, Sweden; now at Max Planck Institute for Biology of Aging, Cologne, Germany). To generate experimental MitoPark mice and littermate controls, the DAT^*cre*^ and Tfam^*loxP*^ mouse strains were first backcrossed to C57BL/6 J mice (The Jackson Laboratory, Bar Harbor, ME), and offspring were selectively mated to generate double heterozygous males (DAT^+*/cre*^; Tfam^+*/loxP*^) and homozygous floxed Tfam females (DAT^+/+^; Tfam^*loxP/loxP*^). The double heterozygous males were then crossed to homozygous Tfam^*loxP/loxP*^ females, resulting in an ~25% yield of MitoPark mice (DAT^+*/cre*^; Tfam^*loxP/loxP*^), along with 25% of littermate controls (DAT^+/+^; Tfam^*loxP/loxP*^). Mice were group-housed in same-sex, standard shoebox cages within a ventilated caging system with *ad libitum* access to food and water. The room was maintained at 26 °C on a 12 hours light/12 hours dark light cycle. All animal care and use was in accordance with the Guide for the Care and Use of Laboratory Animals, 8^th^ edition (NRC). The procedures for all animal experiments were reviewed and approved by the IACUC of the University of Texas Health Science Center at San Antonio.

### Genotyping

DNA was extracted from mouse tail tips by digestion in buffer containing 10 mM Tris-HCl, 50 mM KCl and 0.1% Tween-20 with proteinase K solution (0.2 mg/ml), incubated at 55 °C overnight. Samples were centrifuged at 15,000 × g for 20 minutes and supernatant was collected as DNA extract. Polymerase chain reaction (PCR) was performed on 0.5μg of DNA. To identify the DAT^+/*cre*^ genotype, two primer pairs were used. The forward primer sequence was 5′-CATGGAATTTCAGGTGCTTGG, and the reverse primer sequences were 5′-CATGAGGGTGGAGTTGGTCAG and 5′-CGCGAACATCTTCAGGTTCT. For the Tfam^*loxp/loxp*^ genotype, two primer pairs were also used. The forward primer sequence was 5′-CTGCCTTCCTCTAGCCCGGG, and the two reverse primer sequences were 5′-GTAACAGCAGACAACTTGTG and 5′-CTCTGAAGCACATGGTCAAT. GoTaq® Green Master Mix (Promega, Madison, WI) was used for a 35-cycle PCR with settings as follows: 95 °C for 30 seconds, 55 °C for 30 seconds and 72 °C for 45 seconds. The PCR products were separated by electrophoresis on 2% agarose gels and visualized under UV light after ethidium bromide staining. Predicted bands in MitoPark mice were two at 310 and 470 base pairs for DAT^+*/cre*^ and one at 437 base pairs for Tfam^*loxp/loxp*^. One band was predicted at 310 base pairs for DAT^+/+^ (wild-type) and one at 437 base pairs for Tfam^*loxp/loxp*^ from control littermates (Suppl. 1a).

### Construction of GDNF lentiviral plasmid and virus preparation

The lentiviral vector containing MSP was based on the design previously described in detail^20^. The reporter gene (GFP) in the original design was replaced with either a rat GDNF cDNA (gene bank no. NM019139, STS 50–685) or human GDNF cDNA (gene bank no. NM000514, STS 201–836) using standard molecular cloning procedures. The resulting construct was sequenced to verify the insertion site.

Viruses were prepared as described previously^19,20,24^. Briefly, plasmid Lenti-MSP-hGDNF (hGDNF), Lenti-MSP-rGDNF (rGDNF), or Lenti-MSP-GFP (GFP) along with packaging plasmids pMDLg/pRRE, pRSV/Rev and pMD.G_2_ were co-transfected into 293 T cells. The culture media containing lentiviral particles were collected at 36 and 72 hours post-transfection, filtered through 0.45-µm pore size filters, and then concentrated 1000-fold by two rounds of ultracentrifugation at 56,000 × g for 2 h, then at 72,000 × g for 1.5 h. The viral pellets were resuspended in StemPro-34 SFM medium (Invitrogen, Carlsbad, CA) and stored at −80 °C.

### Validation of GDNF lentiviral vectors

Plasmid hGDNF, rGDNF and GFP were individually transfected into murine macrophage cell line RAW 264.7 (ATCC, Rockville, MD). The culture media containing GDNF produced by RAW 264.7 cells were collected at 48 hours post-transfection, and the GDNF concentrations were measured by enzyme-linked immunosorbent assay (ELISA). The cytotoxicity of the neurotoxin 1-methyl-4-phenyl-1,2,3,6-tetrahydropyridinium ion (MPP^+^) and cell viability were measured as described^76,77^. Briefly, human SH-SY5Y neuroblastoma cells (ATCC, Manassas, VA) were cultured at 0.5 × 10^5^ cels/well in 96-well plates and treated with hGDNF, rGDNF, or GFP medium, respectively, from transfected RAW 264.7 cells for 24 hours. Additionally, a control group of SH-SY5Y cells were cultured without hGDNF, rGDNF, or GFP treatment. All wells of SH-SY5Y cells were then treated with 300 µM MPP^+^ (Sigma, St Louis, MO) for 24 hours. Cell viability was determined by colorimetric MTT (tetrazolium) assay.

### Lentiviral transduction and bone marrow cell transplantation

Male and female MitoPark mice at 18 weeks of age were randomly divided into three groups with 12 animals per group. Group 1 received GFP transplantation. Group 2 and 3 received hGDNF and rGDNF transplantation, respectively. Group 4 was age-matched non-MitoPark littermates received GFP transplantation – serving as wild type normal controls. In some experiments, rGDNF group was omitted since hGDNF had shown a slightly higher neuroprotective effect. We wanted to focus on hGDNF which is more clinical relevant. Donors were 12-week old syngeneic non-MitoPark mice. The procedure for lentiviral transduction and bone marrow cell transplantation (BMT) was described previously^78^. Briefly, donor mice were injected four days before bone marrow harvest with 150 mg/kg of 5-fluorouracil. Bone marrow cells were harvested from the tibias and femurs of donor mice by flushing the bones and then passed through a 100 μm nylon mesh cell strainer (BD Biosciences, San Jose, CA) to generate single cell suspensions. The flushing medium was StemPro-34-SFM (GIBCO Invitrogen) supplemented with 2 mM L-glutamine, 100 IU/ml penicillin, 100 μg/ml streptomycin, and 5 units/ml heparin. Bone marrow-derived stem cells were enriched in the lymphocyte medium using density-gradient separation. After washing, enriched bone marrow-derived stem cells were pre-stimulated overnight in StemPro-34-SFM supplemented with 2 mM L-glutamine, 6 ng/ml of murine IL-3, 10 ng/ml of human IL-6, 10 ng/ml of murine IL-1, and 100 ng/ml of murine stem cell factor (PeproTech, Rocky Hill, NJ). The next day the harvested cells were centrifuged and the pellet was resuspended in 800 μl of concentrated viral supernatant supplemented with the aforementioned growth factors. Infections were performed on RetroNectin- (Takara, Otsu, Japan) coated plates for 6 hours in the presence of 4 μg/ml of protamine sulfate (Sigma, St Louis, MO). Prior to BMT, the four groups of recipient mice were irradiated with 950 cGy using a cesium γ-source with head protection by a lead tube (Suppl. 6), and then 3 × 10^6^ transduced cells were injected via tail vein.

### Behavioral Testing

For all behavioral testing, mice were acclimatized to the testing room for at least 1 h prior to the start and the testing were performed by lab technicians blinded to the mouse group information. Horizontal, vertical locomotor activities and fine activities including grooming, exploration on four limbs and sniffing were measured using the Photobeam Activity System (San Diego, CA) before and after BMT in each experimental group, according to the manufacturer’s protocol. The mice were individually placed in a clear polycarbonate testing cage (18 cm W × 29 cm L × 12 cm H) with approximately 0.5 cm of corncob bedding lining the floor. The horizontal/vertical locomotor activities and fine activities were recorded for 60 minutes and assessed in 10-minutes time-bins. Forelimb motor performance was assessed using the stepping test as previously described^44^. Briefly, the stepping test was performed on an open table (1.2 m in length) and each trail was recorded using a digital video camera. First, the animal was allowed to settle at one edge of the table with all limbs touching on the table for 2 seconds. Next, the hind legs were lifted up by pulling up the tail leaving only the forepaws touching the table. The animal was pulled backwards for a distance of 1 m in 5 seconds until the other edge of the table was reached. Nesting was assessed as previously described^79^. Briefly, the mice were individually housed in a clean plastic cage with approximately 2 cm of corncob bedding lining the floor. Two hours prior to the onset of the dark phase of the lighting cycle, a piece of pre-weighed 51 mm-square × 5 mm-thick Nestlet™ cotton pad (Nestlets™, Ancare Corp., Belmont, New York) was placed on the floor of each cage. The cages were inspected 24, 48 and 72 h later for nest construction according to Deacon’s five-point rating scale method^80^ (Fig. 3a) and the remaining intact cotton pad was weighed at the same time. Sucrose preference was measured as previously described^81^. Briefly, the mice were individually housed in a clean plastic cage with approximately 2 cm of corncob bedding lining the floor and allowed *ad libitum* access to drink from two identical volumetric bottles containing either water or 1% sucrose solution. The positions of the two bottles were switched daily and the bottles were weighted to measure the water or sucrose solution consumption every day for 4 days. Sucrose preference was calculated using the following formula:

$$ \% \,{\rm{sucrose}}\,{\rm{preference}}=[({\rm{sucrose}}\,{\rm{solution}}\,{\rm{intake}})/({\rm{water}}\,{\rm{intake}}+{\rm{sucrose}}\,{\rm{solution}}\,{\rm{intake}})]\times 100.$$

### Flow Cytometry

Peripheral blood was collected from GFP-transplanted MitoPark mice at eight weeks-post BMT. After erythrocytes were lysed with Red Blood Lysing Buffer (Sigma, St Louis, MO), cells were labeled with APC-conjugated CD11b monoclonal antibody (BD Pharmingen, San Jose, CA) at a concentration of 0.4 µg per 10^6^ cells and analyzed by flow cytometry (BD FACSCablibur System, BD Bioscience, San Jose, CA) for GFP expression. The peripheral blood samples collected from wild-type C57BL/6 J and GFP transgenic mice were used as negative and positive controls, respectively.

### Tissue Processing and IHC

Mice were anesthetized with ketamine hydrochloride solution (Butler Schein, Dublin, OH) and perfused transcardially with 20 ml of phosphate-buffered saline, pH 7.4 (PBS) followed by an equal volume of 4% paraformaldehyde solution. Brain, heart, pancreas, kidney and testes were dissected out and fixed in the same fixative overnight at 4 °C. The brains were cryo-protected in sequential 10% (2 h), 20% (2 h) and 30% sucrose in PBS solution overnight, while other tissues were transferred to 70% ethanol. The brains were embedded in Tissue-Tek OCT compound (Sakura Finetek USA, Torrance, CA) and processed for cryosectioning at 30 µm thickness in the coronal plane. Other tissues were embedded in paraffin blocks and cut to 4 µm sections for hematoxylin and eosin (H&E) staining. Four series of slides containing every fourth section were prepared for SN, whereas six series of every sixth section were prepared for striatum. Anatomical landmarks were determined according to Paxinos and Franklin. The brain sections were treated with 1% bovine serum albumin in PBS containing 0.3% Triton X-100 for 30 minutes and then incubated with rabbit anti-TH (Millipore, Billerica, MA) at 1:1,000 dilution overnight at 4 °C. The sections were rinsed with PBS and incubated with biotinylated goat anti-rabbit secondary antibody (1:100) for 1 hour, followed by avidin-biotin peroxidase complex (ABC Elite Kit, Vector Laboratories, Burlingame, CA) at room temperature for 1 hour. The chromogen was either 3-amino-9-ethyl carbazole (AEC Chromogen Kit, Sigma) or 3,3′-diaminobenzidine tetrahydrochloride (Liquid DAB Substrate Kit, Invitrogen (Carisbad, CA). DAB-stained midbrain sections were counterstained with cresyl violet and used for stereology. For immunofluorescence staining, the sections were first incubated with either TH antibody or rabbit anti-Iba 1 antibody (1:1,000; Wako Diagnostics, Richmond, VA), followed by Alexa Fluor 568 (1:100; Invitrogen) as secondary antibodies. The images were captured with a fluorescent microscope (Nikon Eclipse TE2000-U, Nikon Instruments, Melville, NY).

### Stereology

The total number of Nissl^+^/TH-immunoreactive neurons in SNpc was estimated, as described before^82^, using the optical fractionator method in combination with unbiased counting rules, an approach that is not affected by either the volume of SNpc or the size of the neurons^83^. Briefly, the reference space (SNpc) in each 30 μm thick midbrain section was outlined at x10 magnification using Stereo Investigator workstation (MicroBrightField, Williston, VT) attached to a Zeiss AxioImager A1 microscope, fitted with a DEI-750 CE video camera (Optronics, Goleta, CA) and a LEP MAC5000 motorized stage controller (Ludl Electronic Products, Hawthorne, NY). Then at random start, Nissl^+^/TH-immunoreactive neurons were counted from every fourth serial section throughout the entire extent of the SNpc using a x63 oil immersion objective (numerical aperture 1.4). Cells were counted only when their nuclei were optimally visualized, which occurred only in one focal plane.

### Optical Density

Optical densities (OD) of the TH^+^ fibers in the striatum were measured from digitized images of every sixth section using NIH ImageJ software (NIH, Bethesda, MD). The measurements were taken from dorsolateral aspects of the striatum that receive the majority of innervation from DA neurons of SNpc. Relative OD of TH^+^ fibers in the striatum was calculated by subtracting the background OD from the measured OD of the dorsolateral aspects of the striatum.

### Quantification of Microglial Engraftment to SN

To demarcate the SN, midbrain sections of GFP-transplanted MitoPark or normal control mice were first immunostained with TH antibody and an identical section was immunostained with Iba-1 antibody, followed by Alexa Fluor 568–conjugated secondary antibody. Images of TH-stained neurons (red channel) and GFP-positive microglia (green channel) were captured separately and merged using Adobe Photoshop CS2 (Adobe, San Jose, CA). All Iba-1 positive microglia and GFP-expressing cells within the SN were counted from five representative sections per animal.

### ELISA assay for GDNF

Plasma was prepared from peripheral blood collected in EDTA-treated tubes. Midbrain tissue containing SN and forebrain tissue containing striatum were dissected out, respectively, in 1.0 mm thick coronal slices using a mouse brain slicer matrix (Zivic Insruments, Pittsburgh, PA) according to the mouse brain atlas. The slices were stored at −80 °C until analysis. The frozen tissue samples were homogenized in tissue lysis buffer containing 137 mM NaCl, 20 mM Tris (pH 8.0), 1% NP-40, 10% glycerol, 1 mM phenylmethylsulfonyl fluoride, 10 μg/ml aprotinin, 1 μg/ml leupeptin, and 0.5 mM sodium vanadate at a tissue concentration of 30 mg/ml and centrifuged at 20,000 × g for 10 minutes at 4 °C. The GDNF concentrations in plasma, SN and striatum were measured using a commercially available GDNF E_max_ ImmunoAsssay System (Promega, Madison, WI), according to the manufacturer’s protocol. The assay detects both human and rodent GDNF.

### Cold Allodynia Testing

A mouse was placed in a clear plastic cage with a wire mesh bottom. A small drop of 100% acetone was gently applied to the mid-plantar surface of the right hindpaw with a fine pipette. The frequency and duration of paw lift in response to acetone (cold) were recorded as previously described^84^. Acetone was applied five times with an interval of 5 minutes between each application. The response frequency was calculated using the following formula:

$$[(\mathrm{number}\,{\rm{of}}\,{\rm{trials}}\,{\rm{with}}\,{\rm{paw}}\,\mathrm{lift})/({\rm{total}}\,{\rm{number}}\,{\rm{of}}\,{\rm{trials}})]\times 100.$$

The duration of paw lift was the cumulative time of paw lift holding during the first 60 seconds after acetone application.

### Statistical analysis

The statistical analysis of the data was performed using GraphPad Prism 5.03 (GraphPad Software, LaJolla, CA) and multiple group comparisons were analyzed by two-way ANOVA, followed by *post-hoc* analyses using Bonferroni post-test. The nonparametric comparison between the groups was analyzed by one-way ANOVA, followed by Tukey’s post-test. Differences among treatment groups were considered statistically significant at *p* < 0.05.

### Data Availability

All our data in this article will be provided upon request.

## Electronic supplementary material

Supplementary information
