# Supplementary material for: GDNF-expressing macrophages mitigate loss of dopamine neurons and improve Parkinsonian symptoms in MitoPark mice
Source: Sci Rep. 2018 Apr 3;8:5460. doi: 10.1038/s41598-018-23795-4 (PMC5882968; doi:10.1038/s41598-018-23795-4)
Supplement: Supplementary file 1 — Supplementary information [file 41598_2018_23795_MOESM1_ESM.pdf]

Supplementary Information

GDNF-expressing macrophages mitigate loss of dopamine neurons and improve Parkinsonian symptoms in MitoPark mice

Cang Chen[1], Xiuhua Li[1], Guo Ge[1], Jingwei Liu[1], K.C. Biju[1], Suzette D. Laing[1], Yusheng Qian[1], Cori Ballard[1], Zhixu He[3], Eliezer Masliah[4], Robert A. Clark[1,5], Jason C. O'Connor[2,5], and Senlin Li[1,2,5]

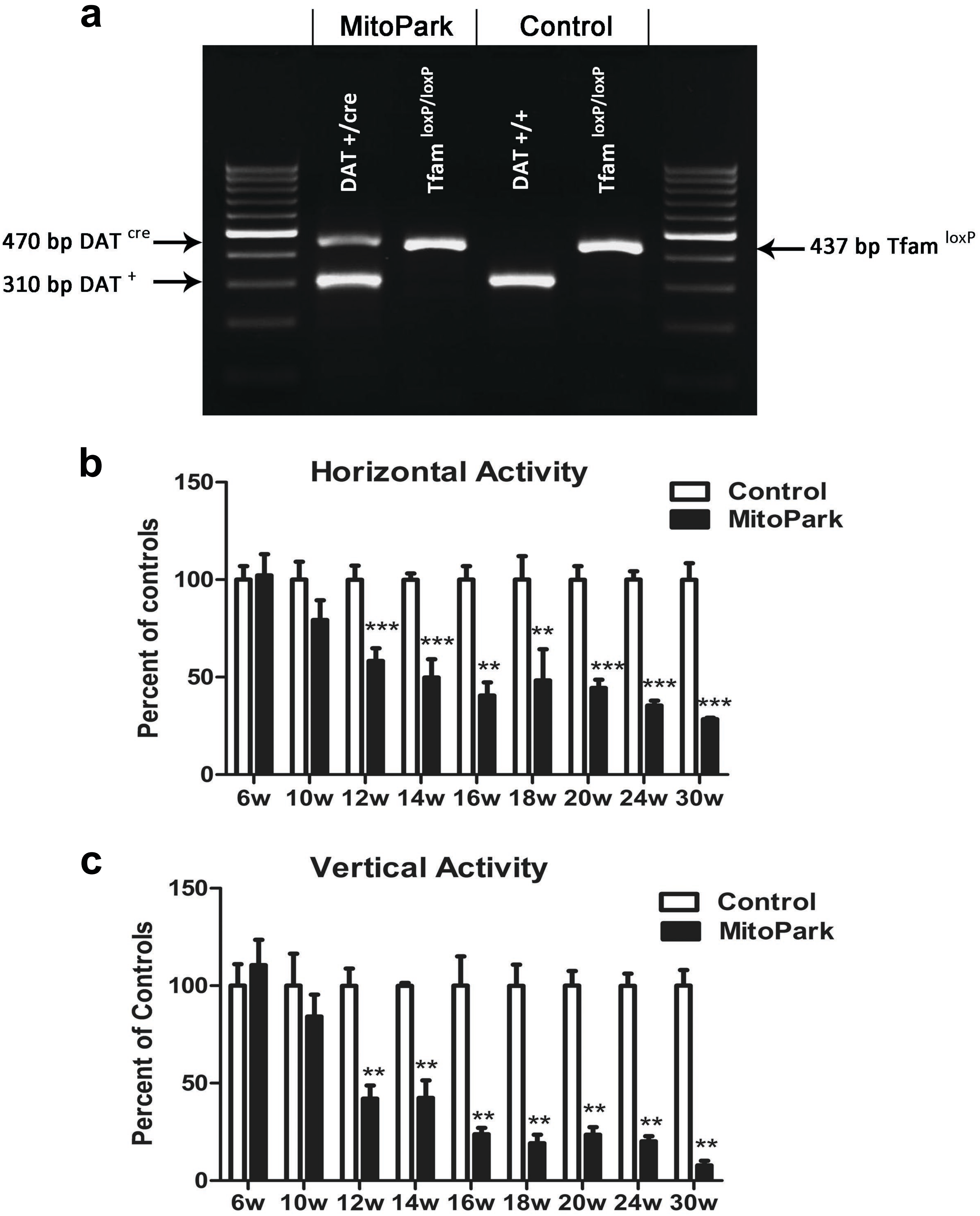
a
MitoPark
Control
DAT +/cre
Tfam loxP/loxP
DAT +/+
Tfam loxP/loxP
470 bp DAT cre
310 bp DAT +
437 bp Tfam loxP
b
Horizontal Activity
Control
MitoPark
Percent of controls
150
100
50
0
***
***
**
**
***
***
***
6w
10w
12w
14w
16w
18w
20w
24w
30w
c
Vertical Activity
Control
MitoPark
Percent of Controls
150
100
50
0
**
**
**
**
**
**
**
6w
10w
12w
14w
16w
18w
20w
24w
30w

Suppl. 1 (Figure S1). Progressive loss of motor function in MitoPark mice. a. Genotype of MitoPark mice and wild type normal control mice. **b.** Horizontal and **c.** Vertical locomotor activities of MitoPark mice as a percentage of littermate control mice at different ages. Both horizontal and vertical activities were tested by the Opto-Max Activity Meter (Columbus Instruments, Columbus, Ohio) according to the manufacturer's protocol, recorded for 60 minutes and assessed at 10-min time-bins. Each bar represents mean ± SEM from twenty MitoPark mice (n = 20) or twenty same-aged wild type normal control mice (n = 20). Significant differences are indicated: **, $P < 0.01$; ***, $P < 0.001$ versus wild type normal control mice.

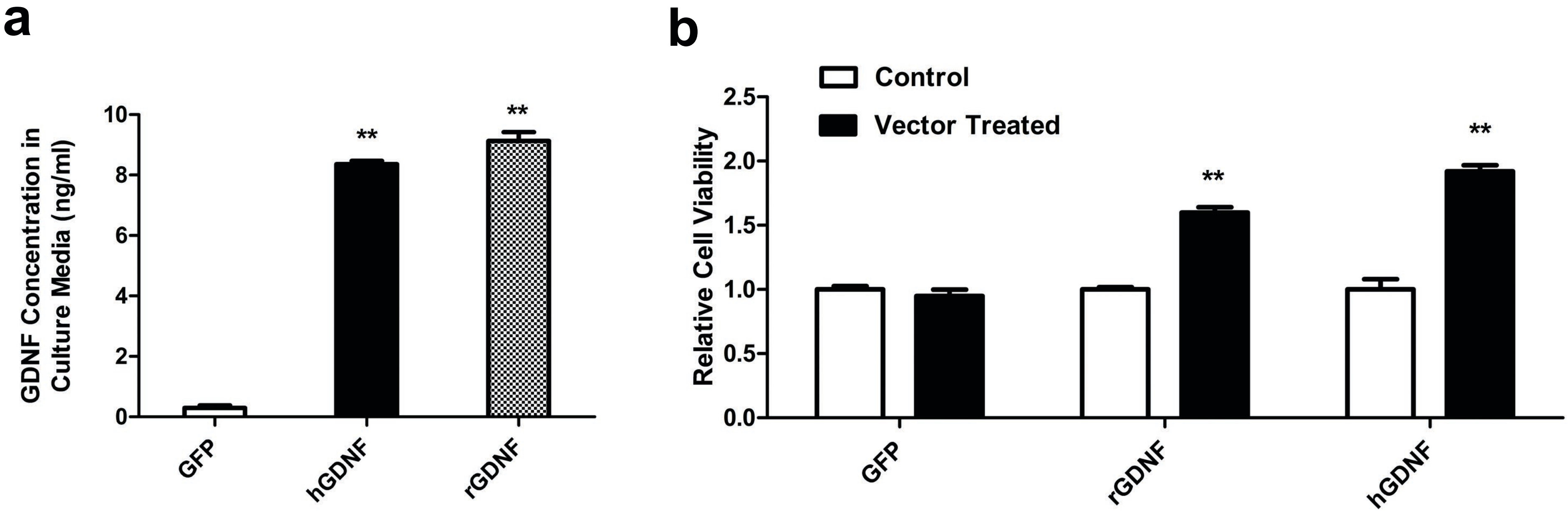
a
GDNF Concentration in Culture Media (ng/ml)
10
8
6
4
2
0
**
**
GFP
hGDNF
rGDNF
b
Control
Vector Treated
Relative Cell Viability
2.5
2.0
1.5
1.0
0.5
0.0
**
**
GFP
rGDNF
hGDNF

Suppl. 2 (Figure S2). Lentiviral vector-expressing GDNF protects the viability of SH-SY5Y cells exposed to MPP^+. a. Plasmid Lenti-hGDNF (hGDNF), Lenti-rGDNF (rGDNF) and Lenti-GFP (GFP) were transfected into murine macrophage cell line RAW 264.7 separately. The concentration of GDNF produced by RAW 264.7 cells in culture medium were measured by ELISA with three replicates per sample. Each bar represents mean ± SEM from six samples per treatment group (n = 6). Statistically significant differences are indicated: **, $P < 0.01$ versus GFP group. **b.** SH-SY5Y cells were pre-incubated with hGDNF, rGDNF and GFP medium separately for 24 hours, their controls were incubated with no vector treated medium, followed by the addition of 300 µM MPP^+ for 24 hours. The cell viability was determined by MTT assay. Each bar represents mean ± SEM from six wells per treatment group (n = 6). Significant differences are indicated: **, $P < 0.01$ versus non-GDNF treated control.

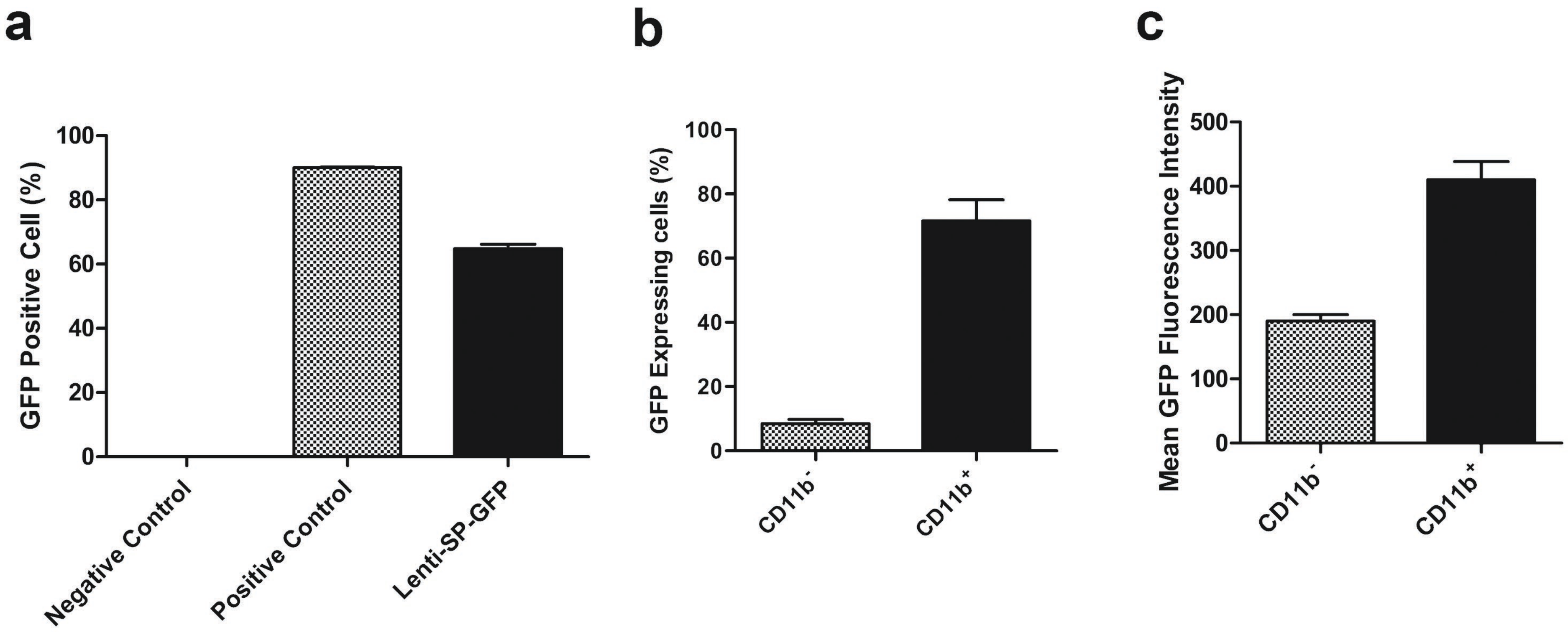
a
GFP Positive Cell (%)
100
80
60
40
20
0
Negative Control
Positive Control
Lenti-SP-GFP
b
GFP Expressing cells (%)
100
80
60
40
20
0
CD11b⁻
CD11b⁺
c
Mean GFP Fluorescence Intensity
500
400
300
200
100
0
CD11b⁻
CD11b⁺

Suppl. 3 (Figure S3). **Expression of transgene in monocytes/macrophages following bone marrow transplantation.** Peripheral blood was collected from GFP-transplanted MitoPark mice at 8 weeks post-transplantation (n = 10). The leukocytes were labeled with APC-conjugated CD11b monoclonal antibodies and GFP expression was analyzed by flow cytometry. The peripheral blood samples collected from wild type C57BL/6J and GFP transgenic mice were used as negative (n = 3) and positive controls (n = 3), respectively. **a.** Percentage of GFP positive cells in the peripheral blood of GFP-transplanted MitoPark mice. **b** and **c.** CD11b positive monocytes/macrophage expressing GFP with high fluorescence intensity.

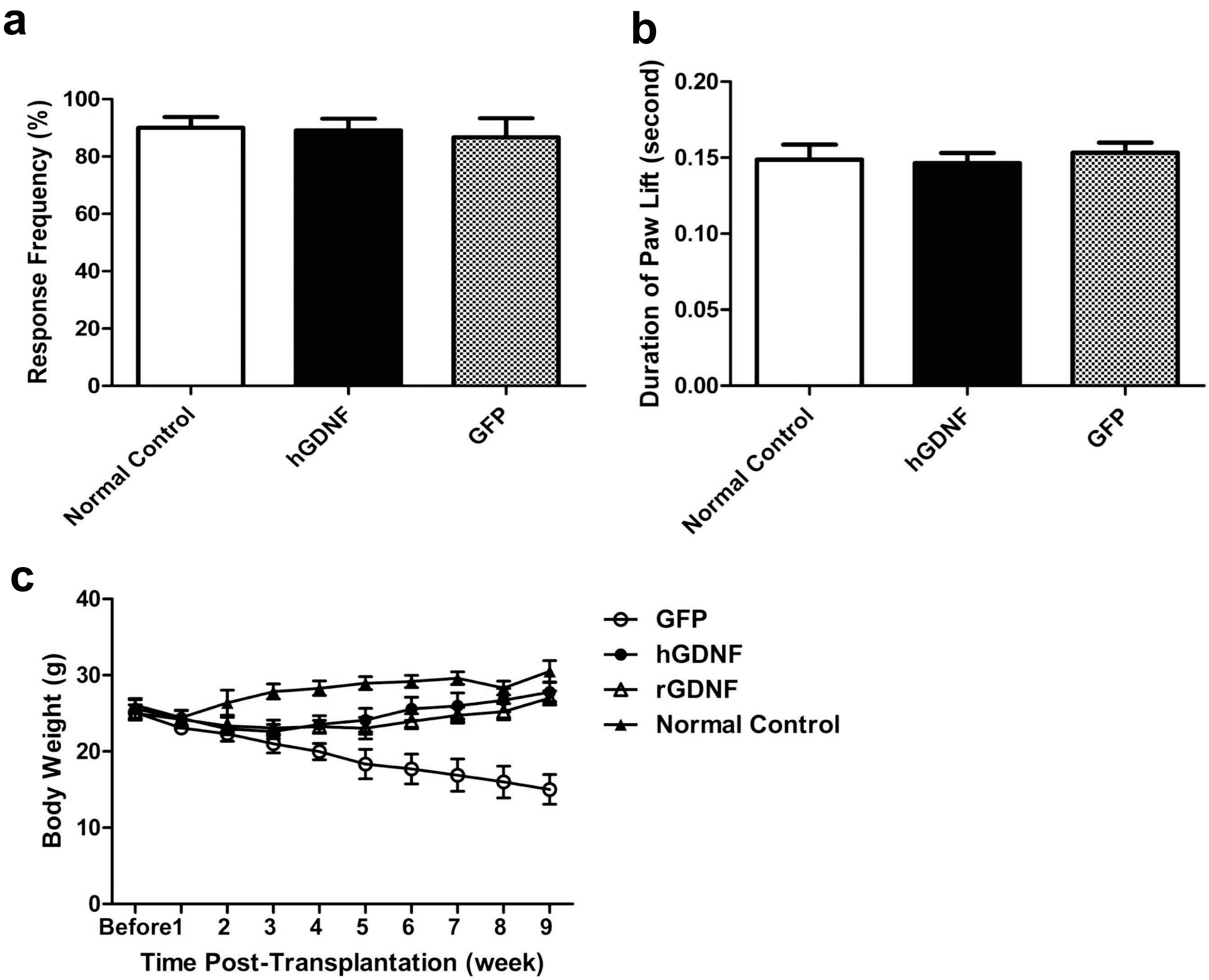
a
Response Frequency (%)
100
80
60
40
20
0
Normal Control
hGDNF
GFP
b
Duration of Paw Lift (second)
0.20
0.15
0.10
0.05
0.00
Normal Control
hGDNF
GFP
c
Body Weight (g)
40
30
20
10
0
Before1
2
3
4
5
6
7
8
9
Time Post-Transplantation (week)
GFP
hGDNF
rGDNF
Normal Control

Suppl. 4 (Figure S4). **Assessment of side effects of macrophage-mediated GDNF therapy. a** and **b.** Cold allodynia testing was performed on hGDNF-, GFP-transplanted MitoPark mice and wild type normal control mice at 8 weeks after transplantation. Each bar represents mean ± SEM of three repeat tests on each individual from ten animals per treatment group (n = 10). **c.** Changes in body weight following GDNF- and GFP-transplantation. As indicated group, each point represents mean ± SEM from ten animals per treatment group (n = 10).

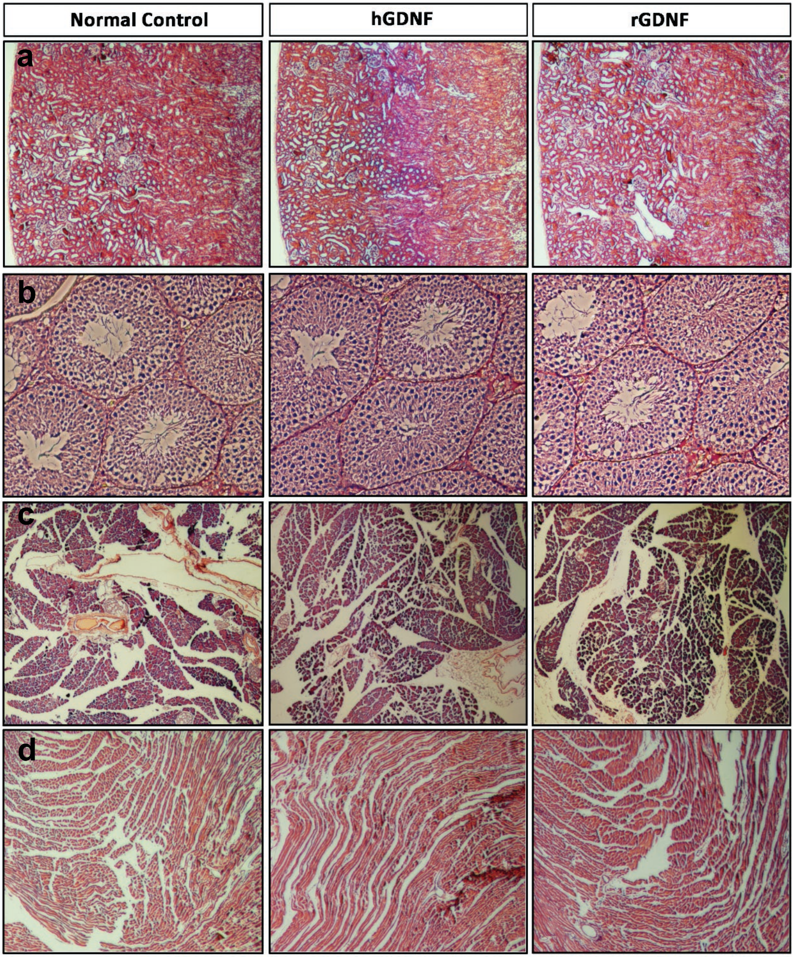
Normal Control
hGDNF
rGDNF
a
b
c
d

Suppl. 5 (Figure S5). **Pathological examination of kidney (a), testis (b), pancreas (c) and heart (d) from GDNF-treated MitoPark mice and GFP-treated normal control mice.** Tissues were collected at 9 weeks after GDNF or GFP transplantation, embedded in paraffin block, cut to 8 μm thick sections and stained with hematoxylin and eosin (H&E). Microphotographs indicating no abnormal tissue morphology were observed in hGDNF- and rGDNF-treated mice.

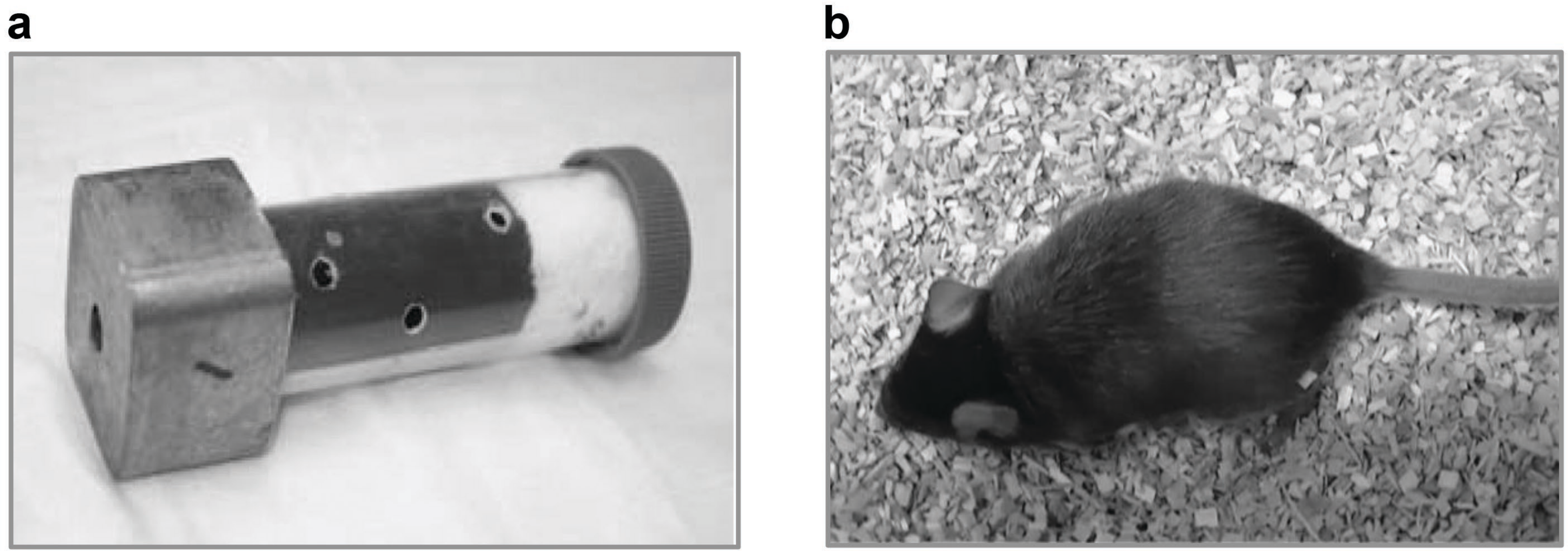
a
b

Suppl. 6 (Figure S6). **Mouse head shielded irradiation. a.** Photograph showing a mouse in a specialized thin-wall plastic tube capped with lead cup prior to irradiation. **b.** The darker hair color above the mouse neck at 9 weeks after irradiation/bone marrow transplantation manifested that the mouse head was protected from irradiation.
